# Micromonolithic Electrochemical Cells for Sustainable Syngas Production from H_2_O and CO_2_

**DOI:** 10.1021/acssuschemeng.4c10889

**Published:** 2025-05-05

**Authors:** Peng Yan, Tao Li, Kang Li

**Affiliations:** † Barrer Centre, Department of Chemical Engineering, Imperial College London, London SW7 2AZ, United Kingdom; ‡ MOE Key Laboratory of Energy Thermal Conversion & Control, School of Energy and Environment, Southeast University, Nanjing 210096, China

**Keywords:** syngas, solid oxide electrolysis, electrochemical reactor, process intensification, process electrification

## Abstract

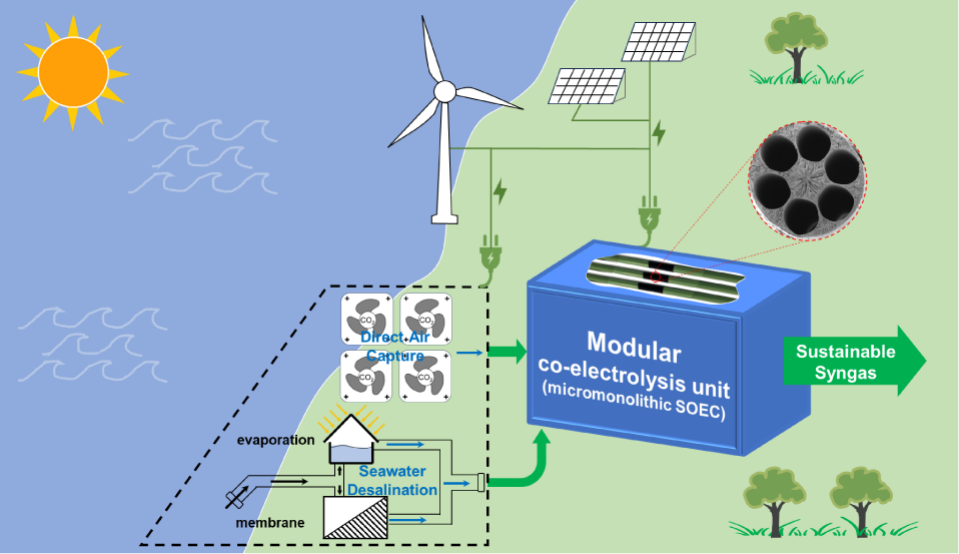

The direct conversion of CO_2_, preferably from direct air capture (DAC), and H_2_O from seawater to syngas by renewable electricity, offers an alternative route toward a sustainable future for the chemical industry. To achieve this ambitious goal, an efficient electrochemical conversion route is preferred. However, high-performance and cost-effective devices for achieving such sustainable production are lacking. Here, we report an innovative micromonolithic solid oxide electrolysis cell (SOEC) device with a productivity of −2.4 A/cm^2^ at 1.4 V and an operational stability of ∼ −1.0 A/cm^2^ (−11.7 A/cm^3^, 4387 N m^3^_syngas_/h/m^3^) for 110 h; this device has an almost 1 order of magnitude greater cost-effectiveness and has substantial environmental benefits compared to conventional tubular and planar designs. The conceptual process design of prospective sustainable electrified syngas production has the potential to achieve 0.1 $/Nm^3^_syngas_ and −0.92 kgCO_2_/kg_syngas_. Moreover, microstructural sensitivity, three-stage degradation mechanism, and mechanical features of the cell are studied to provide deep insights.

## Abstract 2

Process electrification of sustainable syngas production via cost-effective and high-performance micromonolithic SOECs. Contributing to UN SDGs 7, 13.

## 1 Introduction

The chemical industry is in a rapid revolution to decarbonization, electrification, and sustainability, which are driven by Net-Zero, renewable energy, and sustainable development goals (SDGs).^1−4^ Due to the rapid timeline, redesigning chemical processes, particularly those involving large amounts of production and high carbon emissions, is extremely critical and pressing.^3^ Syngas is a typical material that acts as a bridge between raw materials and valuable fuels, chemicals, fertilizers, etc. (Scheme S1). Today, the global market size is approximately 261 million Nm^3^/h, the projected annual growth rate is 11.45%,^5^ and carbon emissions are more than 1.5 kgCO_2_/kg_syngas_.^6,7^ However, it is mainly produced by coal gasification and methane steam reforming today and some research on these processes are still going on.^8−12^ Revolutionizing the syngas production process through electrochemical reactions, which use sustainable nonfossil feedstocks (CO_2_, H_2_O) and are powered by renewable electricity,^13,14^ is deemed to be the key enabling technology for the future of the chemical industry.

Electrochemical reaction technologies for direct syngas production from CO_2_ and H_2_O mainly consist of polymer-based low-temperature electrocatalytic reaction systems (20–200 °C) and ceramic-based high-temperature electrochemical reaction systems (500–900 °C) with a solid oxide electrolysis cell (SOEC) as its core element.^15^ Recent years have witnessed rapid development in low-*T* electrocatalytic reaction systems, including ion exchange membranes, advanced catalysts, and various reactor designs.^16^ However, it still suffers from high overpotential, CO_2_ precipitation, H_2_ crossover, and noble metal demand and has a long way to go in industrial practice.^16,17^ SOECs, as illustrated in Scheme S2, can complement the above technical challenges with demonstrated high energy efficiency, nonnoble metal catalysts, high kinetics, and well-developed materials, and bridge the time gap due to potentially quicker deployment toward practical applications.^15,18^ Recent years have witnessed some remarkable advances in the use of SOECs for syngas production, including the development of new perovskite electrode materials, the control of syngas H_2_/CO ratios, and the understanding of coelectrolysis mechanisms.^19^ However, there remain some major challenges in device design and prospective process development, for which limited attention has been given until recently.

For SOEC device design, recent decades have experienced substantial advances, including commercialization-ready planar design and promising tubular design,^20,21^ where tubular design results in better performance in fast start-up, easy sealing, and excellent thermal cycling stability.^21^ However, moving forward to cost-effective, high-performance devices and promoting rapid SOEC technology deployment in a decentralized manner aligned with the distributed sources of CO_2_ and renewable electricity, disruptive scientific breakthroughs in device design are still critical and urgently needed in view of the gradual maturation of materials development and the limitations of current devices. For this reason, Pirou et al. revolutionized conventional planar design and proposed a novel microscale monolithic SOEC design.^22^ Kelsall et al. reinnovated the conventional planar design to have many microscale pillared structures by using ink-based 3D printing.^23^ Tarancón et al. leveraged the 3D printing technology to produce corrugated planar design.^24^ All of these new designs can achieve several times greater volumetric power density and specific power than those of conventional planar designs. Tubular devices are considered to be superior to planar devices in several aspects, but high internal resistance, including mass transfer and electrical conductivity, through tubular walls has been the key hurdle in improving performance.^21^ Tong et al. took use of 3D printing to fabricate the tubular design and can achieve the large-area production.^25^ CoorsTek company^26^ and Sun et al.^27^ used the paste extrusion technique to make the tubular design. However, the tubular cells made by all these techniques are limited to the large tubular diameter and thicker wall. The microtubular design with versatile microstructures can present some intrinsic advantages, e.g., larger volumetric surface area, thin wall for better mass transfer or resistance, and so on. Our research group has developed microtubular monolithic electrochemical cells based on our expertise in inorganic hollow fiber membranes and has demonstrated their versatility^28^ and ultrahigh performance in solid oxide fuel cells (SOFCs), for example, SOFCs with H_2_ fuel and N_2_O oxidants,^29^ SOFCs with low-value waste methane fuel,^30^ synchrotron XRD-CT approaches for real-time in situ monitoring,^31^ and multiphysics modeling to explain the underlying mechanisms of their intensified performance.^32^ Compared to the single-channel microtubular design, the micromonolithic design with several channels has advantages in higher mechanical strength due to the geometrical cross section, etc., and thus high potentials in industrial scale-up. However, limited efforts have been made to determine its value in high-temperature electrolysis processes, which this article aims to address.

In addition to the design of electrochemical cells, prospective process development has the same importance to inspire practical applications.^33^ By taking into account the upstream and downstream regions and putting the innovative device in the central position at the right places (e.g., the seaside with abundant sources in sustainable feedstocks and electricity), the well-proposed sustainable process design together with comprehensive technoeconomics and environmental analysis can move a further step of cell design at the device level to a more practical system level.^34,35^ Furthermore, comparative analysis between innovative cell design and conventional cell design in the same context can eliminate the different standards in cost estimation or environmental assessment and thus is crucial to fully understand the innovation of a disruptive technology advance.^6^ Unfortunately, less attention has been given to this aspect of newly developed technology.

To address the above-mentioned issues, herein, we focus on syngas production to design innovative micromonolithic SOEC devices, develop corresponding sustainable processes, and make systematic comparisons to conventional designs. While harnessing the technology-ready seawater desalination to H_2_O and DAC to CO_2_, we innovate the electrolysis cell design to significantly improved performance and conceive the conceptual chemical process design. This enable us to achieve the potentially economic feasible and environmental benign syngas production route, by which we bypass the under-debate direct seawater electrolysis^36^ to supply H_2_ for atmospheric CO_2_ capture and conversion.

## 2 Experimental Section

All of the materials used in the experiments are commercially available. SOEC cells were fabricated by a combined controllable phase inversion and sintering method and tested in a home-built experimental setup (mainly consisting of mass flow controller, furnaces, electrochemical workstation, and gas chromatography). Material properties, geometrical morphology, gas-tightness, and mechanical strength are characterized by X-ray diffraction (XRD), scanning electron microscopy (SEM), home-built setup, and a tensile tester, respectively. All of the details can be found in the Supporting Information.

Technoeconomics (TEA) for sustainable syngas production via innovative micromonolithic cells was conducted by following the chemical process design principles, and life cycle assessment (LCA) was performed by following the LCA methodology with a focus on the gate-to-gate scope. For more details, please read the Supporting Information.

## 3 Results and Discussion

In Section 3.1, comprehensive manufacturing and performance studies at the device level are presented; namely, a micromonolithic SOEC is fabricated, and systematic evaluations are conducted, including investigations of the electrochemical performance, microstructure sensitivity, mechanical behavior, long-term stability, and degradation mechanisms. Section 3.2 presents rich information at the process level, where a sustainable electrified syngas production process is developed and evaluated via techno-economics and gate-to-gate life cycle assessment. In Section 3.3, a systematic comparison between a novel micromonolithic design and a conventional planar and tubular design was conducted by utilizing cross-disciplinary knowledge of the membrane module design through analogy.

### 3.1 Section I. Innovative Micromonolithic SOEC Device

To provide a systematic and in-depth understanding of innovative cell design, this section not only discusses micromonolithic cell design, cell manufacturing, and electrochemical performance evaluation in various configurations, which are important for fundamental design, but also reveals the long-term stability and performance degradation mechanism and mechanical strength and suitable scale-up strategies, which are critical for advancing proof-of-concept technology for practical implementation.

#### 3.1.1 Innovative Design and Cell Manufacturing

**Scheme 1:**
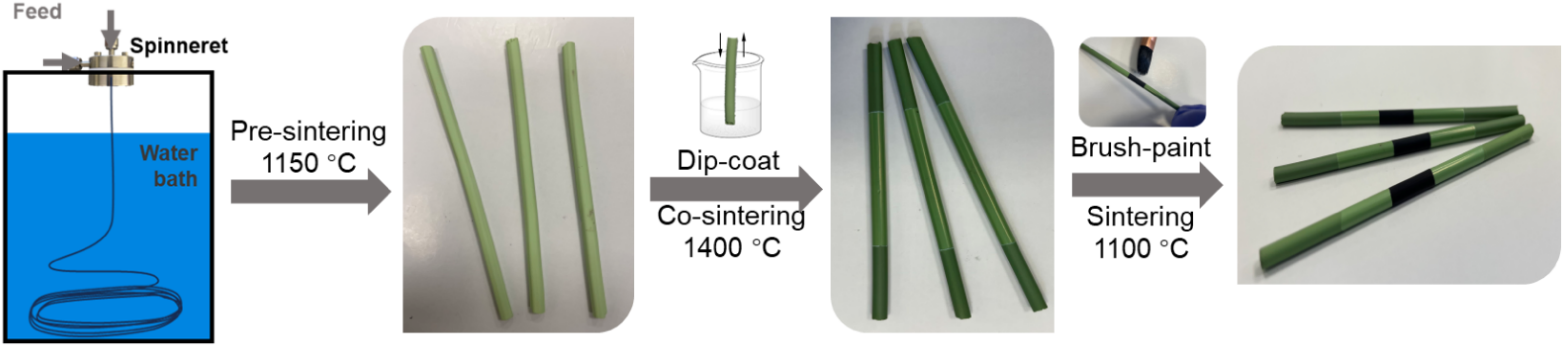
Combined Controllable Phase Inversion and Sintering Method to Micromonolithic SOEC

The SOEC device has a micromonolithic design and consists of 6 separate submillimeter gas flow channels. Scheme 1 presents the micromonolithic SOEC cell manufacturing process. Cell manufacturing is based on combined controllable phase inversion and sintering methods, including micromonolithic spinning to prepare a YSZ8 (8 mol % Yttiria-stabilized zirconia, characterization shown in Figure S2)-NiO (characterization shown in Figure S2) electrode precursor, presintering for initial mechanical strength at 1150 °C, dip coating and cosintering for robust and gastight electrolytes (YSZ8|YSZ8-NiO) at 1400 °C for 6 h, and final brush painting with LSM ((La_0.8_Sr_0.2_)_0.95_MnO_3-δ_) containing ink and sintering at 1100 °C to the full cell (LSM/LSM-YSZ8|YSZ8|YSZ8-NiO). The porosity of the full sintered sample after H_2_ reduction is 29.3% as characterized by mercury intrusion porosimetry. It is important to mention that the reasons for selecting YSZ8-NiO as the electrode materials are that YSZ8-Ni after H_2_ reduction is formed and very active for CO_2_ and H_2_O activation and YSZ8-Ni presents high electrical conductivity due to Ni and oxygen ion conductivity due to YSZ8. The other studied electrode materials are Cu replacing Ni or all perovskite etc. One of the key disadvantages of Cu (1083 °C) is its much lower melting point than Ni (1453 °C) and this causes stability issues in both high-temperature treatment for full cell preparation and high-temperature electrolysis operation.^37,38^ Additionally, all perovskite electrodes normally present lower electrical conductivity and thus low overall electrolysis performance.^38^ During cell manufacturing, many controlling parameters (e.g., recipe, sintering temperature, and coating operation) can affect the final cell performance, and each of the influential parameters can be tuned to prepare various cells with different configurations, e.g., composition, microstructure, and dimension. Overall, this manufacturing method offers many opportunities for controllable tuning, which is important for optimizing the design and mechanistic studies of SOECs.

**Figure 1:**
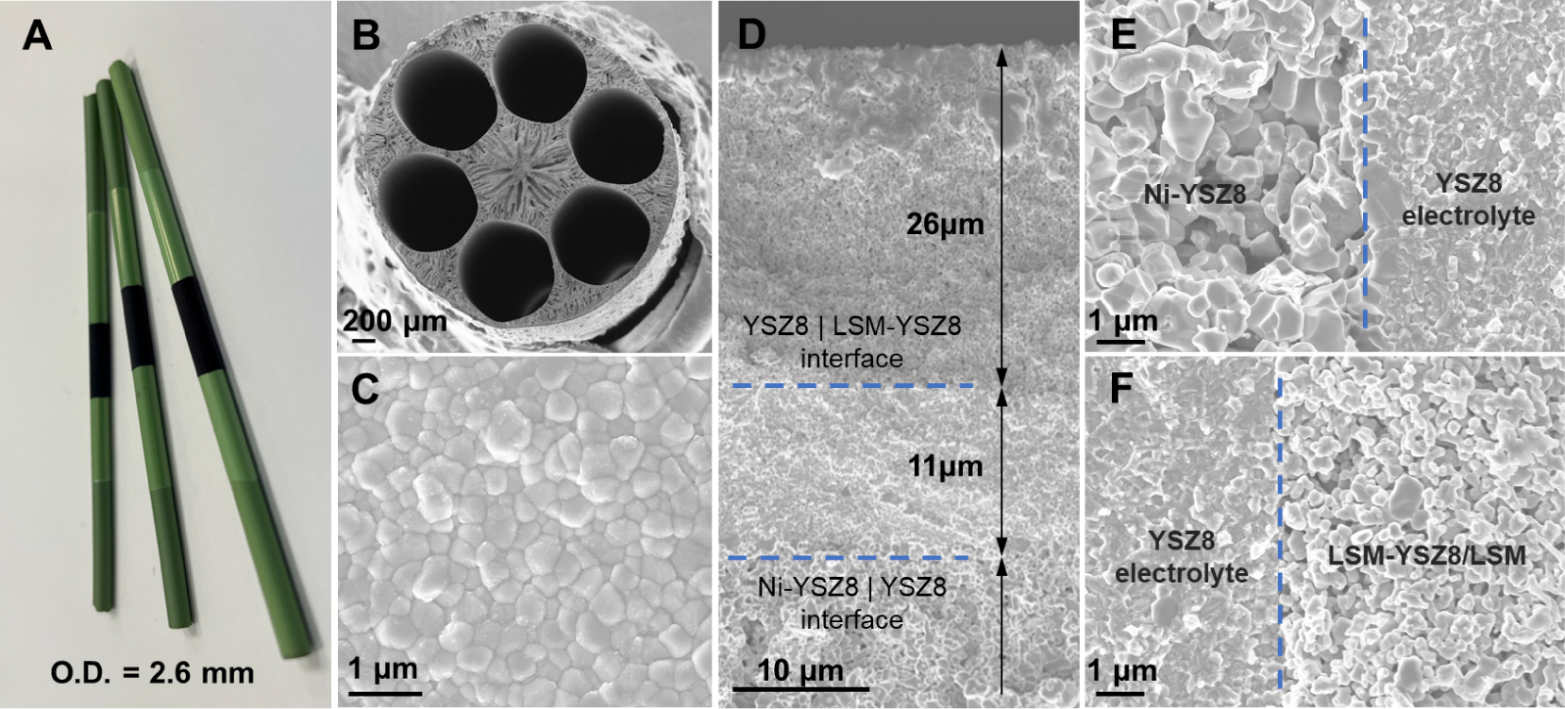
Micromonolithic SOEC device. (A) Optical image of 6-channel hollow fiber cells and (B) their cross-sectional microstructure, (C) electrolyte surface, (D) triple-layer microstructure, and (E,F) interfaces.

Figure 1 presents an optical image and the morphology of the as-prepared micromonolithic SOEC (default configuration #1). The as-prepared cell had an outer diameter of 2.6 mm and a diameter of 0.9 mm for each gas flow channel, as shown in Figure 1B. In principle, the submillimeter gas flow channel enables better external gas mass transfer than conventional designs with several millimeter channels. Figure 1C and the results from the gas-tightness experiment shown in Figure S3 prove sufficient densification of the electrolyte layer. Figure 1D–F shows the clear and well-connected interfaces inside the cell without any delamination. Moreover, it is worth mentioning that the cell used for SEM morphology analysis was the spent cell after the long-term stability test (presented in the next section). As a comparison, the as-prepared cell interfaces are shown in Figure S4, and there is no difference observed between the spent one and the original one. These factors are of critical importance for practical applications because delamination is one of the key factors leading to fast degradation in conventional designs.

#### 3.1.2 Performance and Microstructure Sensitivity

To reveal the electrochemical performance, particularly coelectrolysis, of the micromonolithic design, systematic experiments are conducted. Figure S5 presents the electrochemical performance and quantitative analysis for default configuration #1. Clearly, the fuel cell mode data demonstrate the excellent gas-tightness of the micromonolithic SOEC device, as observed in the OCV (open circuit voltage) values around 1.18 V (vs theoretical OCV ∼ 1.20 V). Moreover, the cell performance indicates an obvious temperature dependence (700–800 °C) and the largest polarization resistance percentage from concentration polarization (i.e., mass transfer resistance) in the low-frequency range (0.1∼25.12 Hz). In the electrolysis mode, the micromonolithic cell presents an excellent current density of −1.8 A/cm^2^ at a thermoneutral voltage (1.4 V), which is higher than that of conventional designs with the same materials.^20^ This proves the pivotal role that device design can play in performance improvement, which is mainly attributed to both the 6-channel monolithic design and the microstructured length scale.

**Figure 2:**
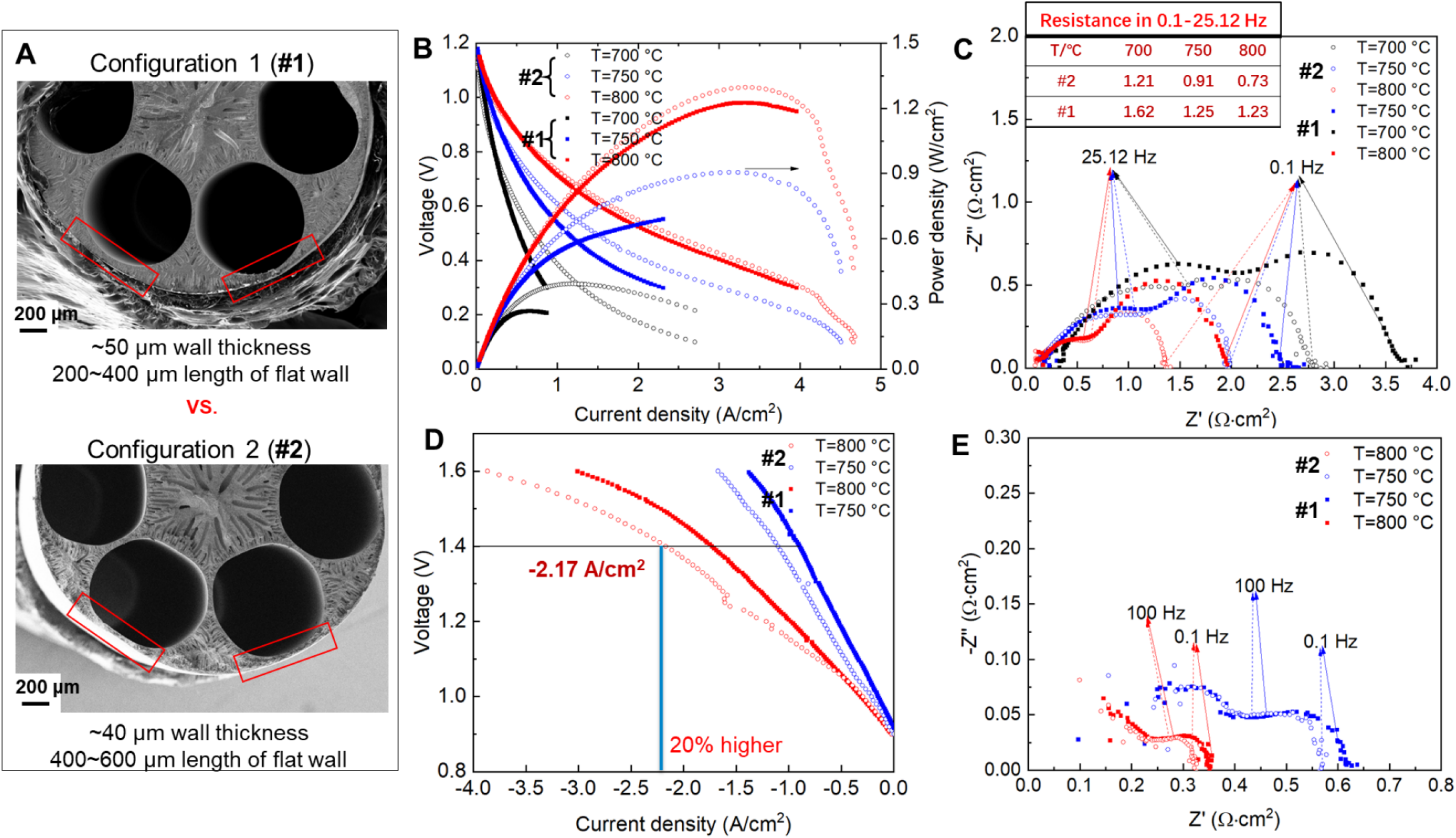
Micromonolithic SOEC cell performance and its microstructure sensitivity. (A) Micromonolithic electrode configurations with different outer wall thickness and flat wall length. (B,C) Comparison of *I*–*V* curve and EIS under fuel cell mode, where inserted tab indicates the mass transfer resistance. (D,E) Comparison of *I*–*V* curve and EIS under electrolysis mode. Complemented by Figures S4 and S5. Feed composition: 45 sccm of H_2_O, 27.5 sccm of CO_2_, 27.5 sccm of H_2_.

To better understand the cell performance and sensitivity to microstructures, two cell configurations were studied for comparison. Figure 2 presents a comparison and quantitative analysis of their electrochemical performances. Obviously, the micromonolithic SOEC can reach a much higher *I*–*V* of −2.17 A/cm^2^ at a thermoneutral voltage for cell configuration #2 (20% higher than that for cell configuration #1), as shown in Figure 2D, which is evidenced by the impedance reduction in Figure 2E. Figure 2A shows that cell configurations #1 and #2 present obvious differences in the outer wall of the gas flow channel but still have almost the same channel dimension (i.e., nearly identical gas flow in the channel). Cell configuration #1 has a thicker and nonuniform channel wall, typically 50 μm in thickness or above and a 200 – 400 μm flat wall structure in length, and its porosity is 29.3%. However, cell configuration #2 clearly has thinner and more uniform channel walls, which are typically ∼40 μm thick and 400–600 μm long, and its porosity is 28.9%. Considering the diffusion, transport, and reaction processes of CO_2_ and H_2_O gas molecules in the gas flow channel through the outer wall structure, as shown in Figure S6, the outer wall thickness and uniformity can potentially significantly affect performance. This can be observed from the *I*–*V* curve under fuel cell mode, as shown in Figure 2B, and it is further proven by the concentration polarization curve (i.e., mass transfer resistance) shown in Figure 2C, where the decrease in mass transfer resistance reaches 30–40% and the EIS curves in the intermediate and high frequency ranges almost overlap. Moving forward, further performance improvement should be achievable by further tuning of microstructures and the reveal of a fundamental microstructure–performance relationship.

#### 3.1.3 Long-Term Stability

**Figure 3:**
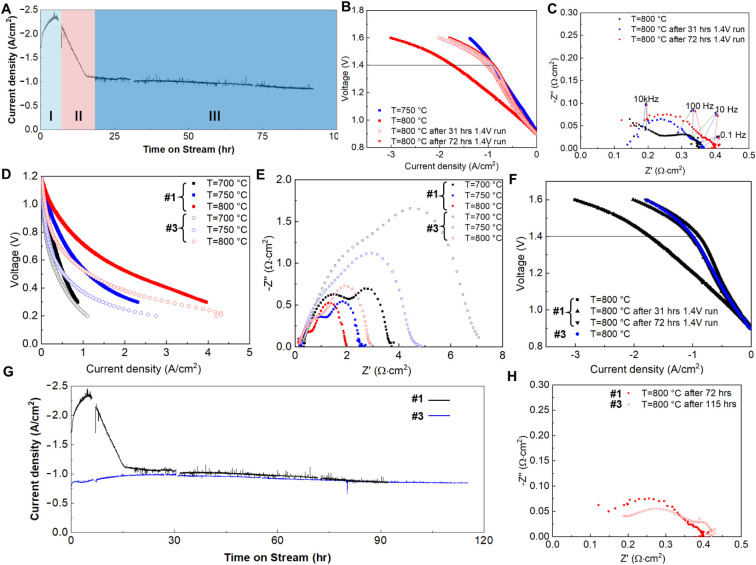
Long-term stability and three-stage degradation mechanism. (A–C) Performance degradation phenomenon and three-stage degradation mechanism under ultrahigh current density (up to −2.4 A/cm^2^) in configuration 1 cell at thermoneutral voltage (1.4 V). (D–G) Configuration #3 presents improved long-term stability by improved design, with (D) slightly lower performance and (E) higher EIS in fuel cell mode and (F) lower electrolysis performance (configuration #3: higher electrolyte sintering temperature 1430 °C for slightly decreasing Ni-YSZ electrode porosity to 28.7% and TPB active sites number, etc., thinner YSZ8-LSM layer ∼ 5 μm (less active sites of oxygen evolution), a bit thicker YSZ8 electrolyte (∼14 μm) as shown in Figure S8). (H) EIS comparison after long-term coelectrolysis tests. Co-electrolysis feed composition: 45 sccm of H_2_O, 27.5 sccm of CO_2_, 27.5 sccm of H_2_. Notes: the disconnection points in (A, G) mean the DI water refilling for the electrolysis system.

To understand the practical importance of micromonolithic SOECs, we studied their long-term stability and performance degradation behavior. Figure 3A shows the long-term operation of cell configuration #1 for up to 100 h at 1.4 V and 800 °C. There are three distinct stages: (1) stage I: a gradual increase in the current density from −1.8 to −2.4 A/cm^2^, which is probably attributed to the activation of both electrodes at the initial stage; (2) stage II: rapid degradation from −2.4 to −1.1 A/cm^2^, which is also clearly revealed by the *I*–*V* curves in Figure 3B; and (3) stage III: relatively stable operation at approximately −1.0 A/cm^2^, which presents almost the same performance as that at 750 °C. As shown in Figure 3C, there was a dramatic increase in the −*Z*’’ resistance in the intermediate frequency range (10 kHz–10 Hz) from 0 to 31 h. Therefore, the underlying mechanism for the fast performance degradation in stage II can be attributed to the rapid decrease in the number of active sites on both electrodes.^39−41^ Interestingly, the rapid decrease occurs in only an extremely short time and becomes stable. To reveal whether fast degradation only occurs during high-current operation, we tested another configuration #1 cell at 750 and 800 °C under galvanostatic conditions, and the results are shown in Figure S7. Obviously, there is no fast performance degradation stage after 30 h. Preliminarily, we can conclude that the fast degradation phenomenon (i.e., a rapid decrease in the number of active sites at both electrodes) is attributed to the ultrahigh current density.

To exclude the effect of temperature (800 vs 750 °C) on the fast degradation behavior of cell configuration #1, we designed cell configuration #3, as shown in Figure S8, by deliberately increasing the overall cell resistance by increasing the electrolyte thickness and decreasing the number of active sites and operating it at 800 °C for long-term stability. Figure 3D–H shows the electrochemical performance and long-term stability of the sample compared to those of cell configuration #1. It is obvious that cell configuration #3 has a lower current density due to its much higher polarization resistance, as indicated in the fuel cell mode in Figure 3D,E. Correspondingly, the electrolysis performance in Figure 3F indicates that cell configuration #3 at 800 °C has a similar coelectrolysis performance as cell configuration #1 after fast degradation at 800 °C. The long-term stability experiment in Figure 3G shows very good stability at approximately −1.0 A/cm^2^ for more than 110 h, which is completely different from that of cell configuration #1, and the generated syngas by coelectrolysis via GC-TCD (typical sampling data are shown in Figure S9) is around 6 sccm with 4.51 sccm H_2_ and 1.54 sccm CO. Figure 3H shows that configuration #3 has better electrode activity than does degraded configuration #1, even though the total resistance is greater. Combined with the operational stability behavior at 750 °C in cell configuration #1, it can be firmly concluded that the rapid degradation behavior is attributed to the ultrahigh current density, and long-term stability can be achieved by careful tuning of the overall cell resistance. This is a large step toward sustaining high performance (∼−1.0 A/cm^2^ at 1.4 V, 800 °C) without sacrificing long-term stable operation.^42,43^ The feed gas conversion is around 8% due to high flow rate, and the future scale-up research is important for achieving the high conversion. The Faraday efficiency is around 93%, which is a bit away from the theoretical 100% in YSZ8 electrolyte.^44^ The reason may come from the accumulated systematic errors from many aspects (gas chromatography calibration etc.).

Importantly, the long-term stability behavior and three-stage degradation mechanism revealed that decreasing the electrolyte layer thickness and increasing the number of active sites or increasing the active site activity in electrodes may be the pitfalls of the endless pursuit before we can ensure that the number of active sites does not decrease under very harsh operation conditions (particularly ultrahigh current density).

#### 3.1.4 Mechanical Features and Scale-Up Strategy

**Figure 4:**
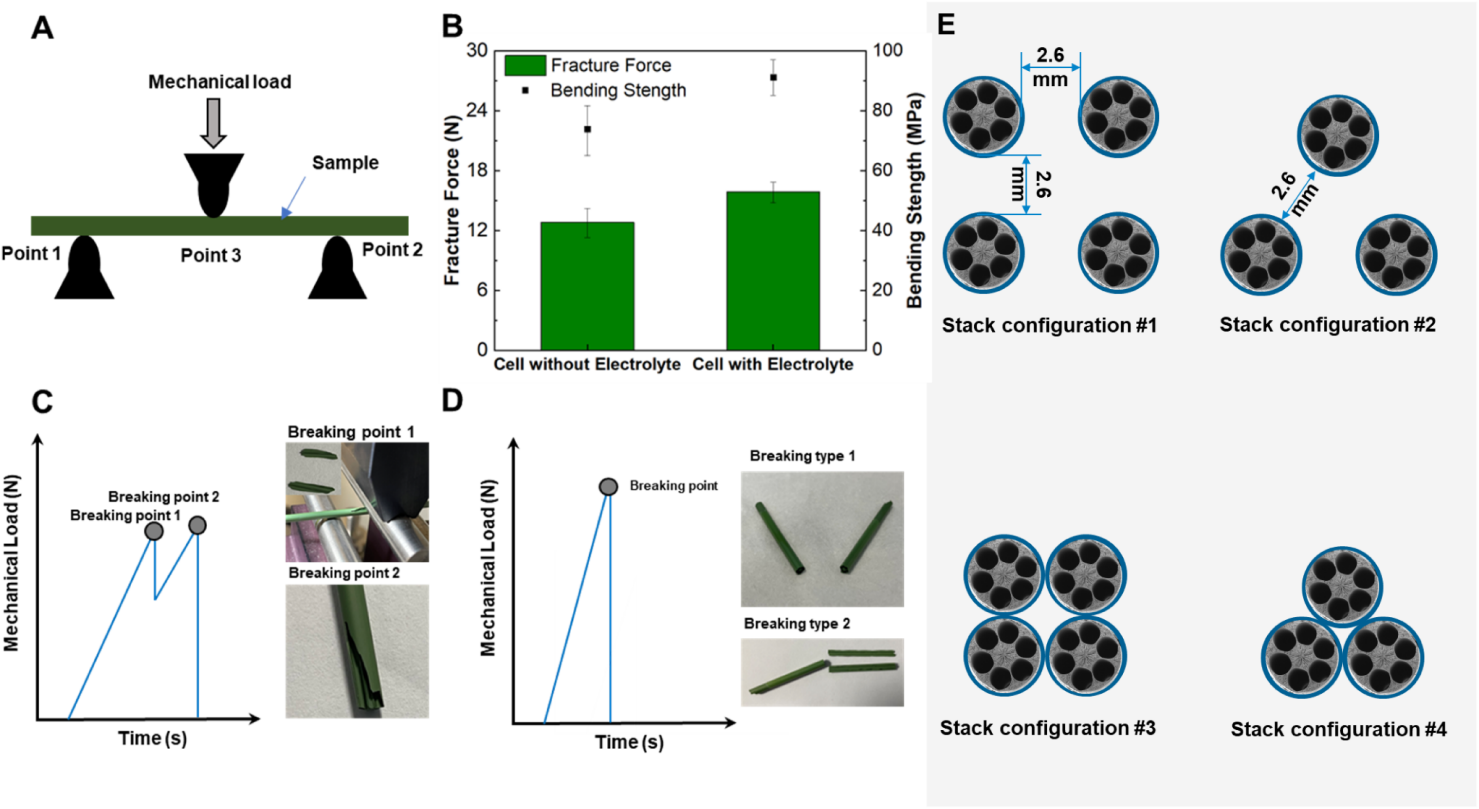
Mechanical strength and breaking behaviors of micromonolithic SOEC cells, and their potential scale-up strategies. (A) 3-point bending method, (B) fracture force and bending strength, breaking behaviors of the cell (C) without electrolyte coating and (D) with electrolyte coating, (E) four typical scale-up SOEC pack configurations. Complemented by Figure S10.

The mechanical strength of the micromonolithic SOEC device is a critical element for practical applications, and an in-depth understanding of its breaking behavior is helpful for further rational design. Figure 4A–D presents the mechanical strength and breaking behavior under the 3-point bending method. In general, the micromonolithic cell with an electrolyte has 24% greater mechanical strength (15.86 N in fracture force, 91.27 MPa in bending strength in Figure 4B), which means that the micromonolithic YSZ8-NiO electrode provides the most mechanical strength, but the electrolyte is critical for strength enhancement considering its very limited thickness. However, the breaking behavior is completely different for micromonolithic cells with or without an electrolyte, as shown in Figure 4C,D. For micromonolithic cells without electrolyte, there are two breaking points: one is the outer wall of the 6-channel, and the other is the central rod, where the central rod has better mechanical strength and thus is the key source of mechanical strength. However, both the outer wall and central rod of the micromonolithic cell with an electrolyte broke at the same time, and the mechanical load at the breaking point was greater than the mechanical load at breaking points 1 and 2 in the micromonolithic cell without an electrolyte. This is attributed to the significant enhancement in the micromonolithic cell with even an 11 μm electrolyte layer. Therefore, it is better to remove the central rod part in the future optimization design of multitubular cells, which will reduce the material cost by more than 35% by calculating its percentage in the effective cross-sectional area, as shown in Figure S11.

Considering the excellent electrochemical performance, long-term stability, and mechanical strength of micromonolithic SOEC devices, scale-up will be the next focus of practical applications. As shown in Figure 4E, we propose 4 kinds of scaling-up configurations together with the parallel-series concept in Figure S12. The prevailing scaleup strategy for tubular devices is to arrange them in rectangular or triangular shapes at some distance, typically one time the characteristic length of the tubular device (2.6 mm in this case), which are stack configurations 1 and 2 in Figure 4E. However, in this case, the mechanical strength should be considered to be greater since the micromonolithic device is slightly more fragile than the conventional tubular device because of the thinner supporting electrode (normally 1 order of magnitude difference in the outer wall dimension). In this case, stack configurations 3 and 4 may be good alternatives because closely bundled structures provide much greater mechanical strength and 3 to 3.6 times greater volumetric productivity (as calculated in Section S3).

To summarize, this novel micromonolithic SOEC has several advantages over conventional planar or tubular designs and has the potential for a modular and compact design for distributed syngas production. A comprehensive understanding of this novel design in terms of the coelectrolysis of CO_2_ and H_2_O is important for rational design and further optimization for general electrolysis applications. However, herein, we adopted only classical materials (LSM, NiO, and YSZ8) to validate the micromonolithic design of the SOEC device. The overall performance, including current density, stability, and mechanical strength, of the micromonolithic SOEC device can be improved if state-of-the-art materials are adopted together with fine control in various aspects. Further efforts should be devoted to integrating micromonolithic design and state-of-the-art materials for a better overall performance.

### 3.2 Section II. Sustainable Syngas Production Process

To move the proof-of-concept novel design one more step closer to practical application, process development is the next key. By placing the coelectrolysis unit with relevant upstream and downstream, we found that the coelectrolysis unit was in the lowest technology readiness level (TRL) stage. Systematic process analysis, including techno-economics (TEA) and life cycle assessment (LCA), will play a pivotal role in providing a deep understanding^45,46^ of the feasibility of innovative design in the early development stage. It can also stimulate and promote rapid and focal research and development in particular directions and accelerate technology deployment.

#### 3.2.1 Conceptual Process Design

**Scheme 2:**
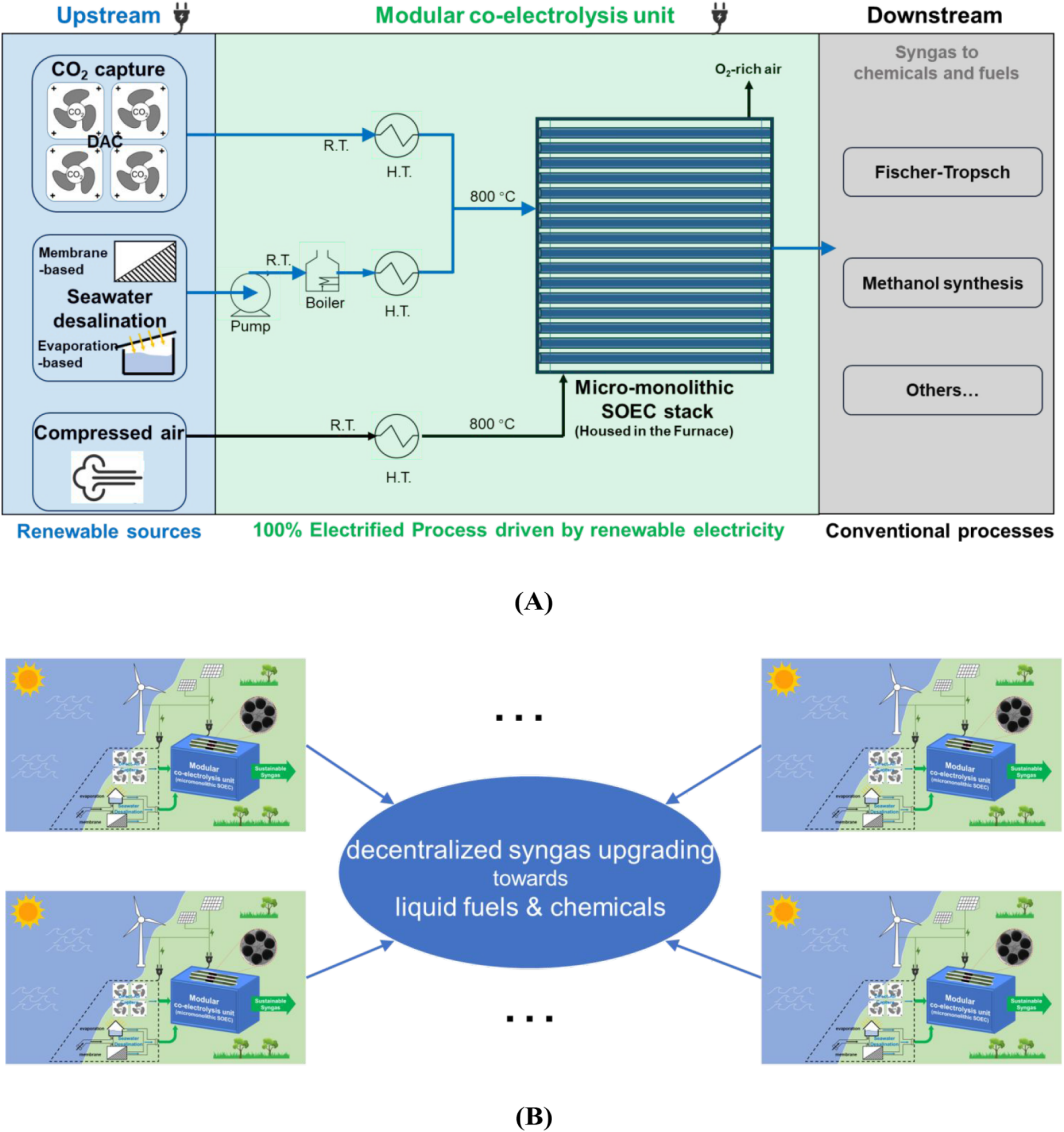
(A) Conceptual Design of Sustainable, Electrified Syngas Production Process with High-Performance, Cost-Effective Micromonolithic SOEC, (B) Envisioned Scenario: Several Modular Syngas Production Units at the Seaside (Enlarged Picture in Figure S13) Are Connected to Larger Decentralized Syngas Upgrading Plant in the Downstream Notes: DAC (direct air capture); R.T. (room temperature); H.T. (heat exchanger).

In the context of the future of the electrified chemical industry, Scheme 2A depicts the conceptual process design of a sustainable, 100% electrified syngas production process. The central part is a modular coelectrolysis unit, which is composed of an SOEC stack based on a novel micromonolithic cell design and complementary heat exchangers, a pump, and a boiler. This central part is assumed to be 100% powered by renewable electricity. The upstream unit, which consists of a CO_2_ capture unit, seawater desalination unit, and compressed air unit, provides renewable sources to the central coelectrolysis unit. The syngas produced in the modular coelectrolysis unit directly goes to the well-established chemical process in the current chemical industry, e.g., Fischer–Tropsch and methanol synthesis, to produce valuable chemicals and fuels. In this way, the conceptual design depicts a fully recyclable (closed-carbon-loop), renewable, sustainable process. Scheme 2B presents the envisioned scenario for integrating several modular syngas production units connected to a larger decentralized syngas upgrading plant. In this scenario, the seaside will be the optimal place because the place intrinsically has abundant feedstocks (water from seawater, CO_2_ from DAC) and abundant renewable electricity to power feedstocks mined from both nature and modular coelectrolysis units. The seaside, as the source place for renewable electricity (RE), possesses the cheapest electricity supply by eliminating the large electricity storage or transmission cost.^47^ Furthermore, decentralized syngas upgrading to liquid fuels and chemicals enables more sustainable development in the broad supply chain. Lastly, it is worthy to point out that the realization of conceptual process will be much easier by supplying CO_2_ from high-TRL capture from power plants rather than DAC and directly using nonsalty water in the land. Instead, we consider a more radical and future-focused conceptual process design with CO_2_ from DAC and H_2_O from seawater from a long-term perspective.

#### 3.2.2 Technoeconomics

**Figure 5:**
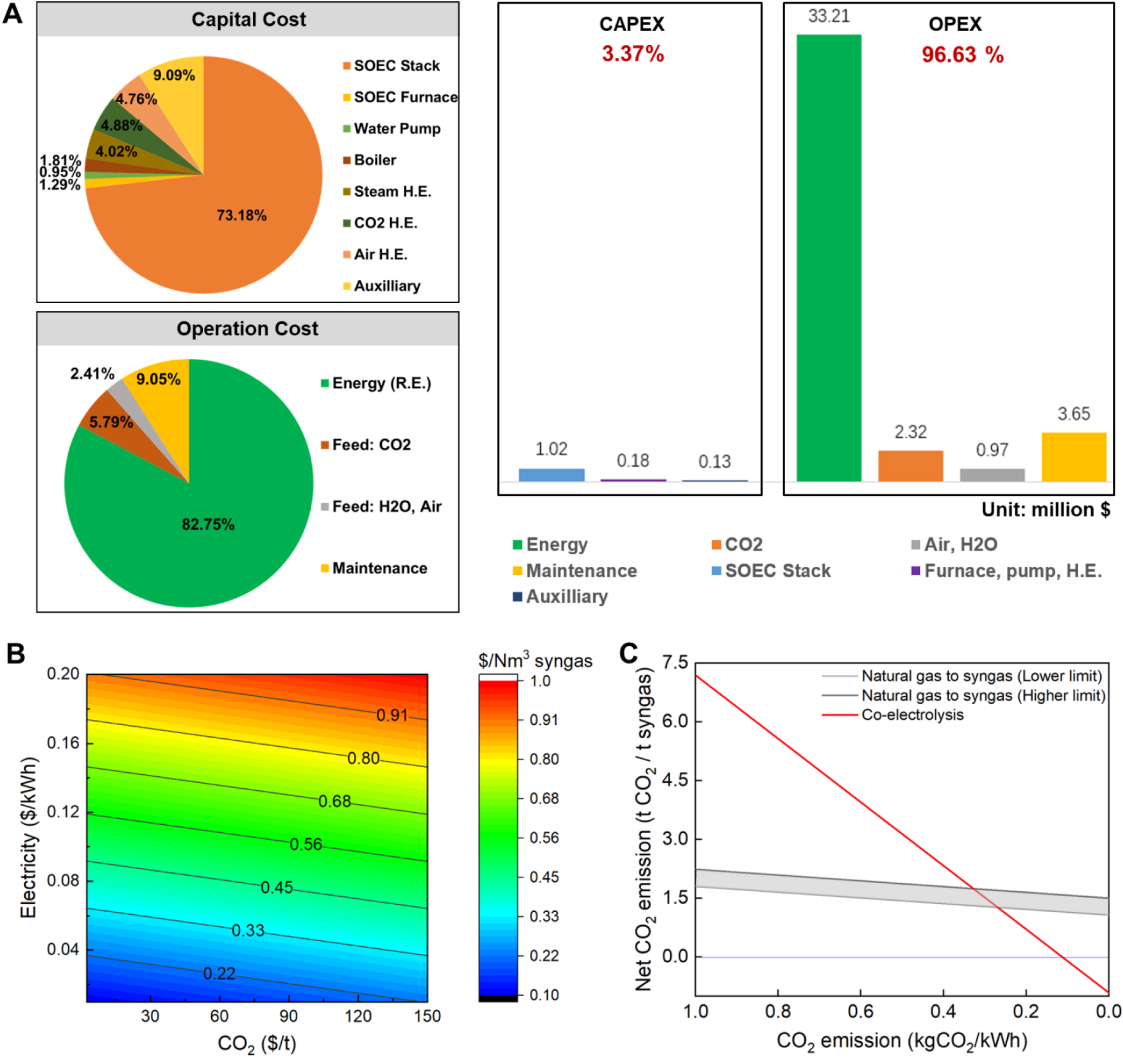
Technoeconomics and gate-to-gate life cycle assessment of the sustainable syngas production process at 1.0 kNm^3^/h syngas productivity. (A) Cost breakdown of CAPEX and OPEX when assuming 75 $ per metric ton in captured CO_2_, 0.20 $/kWh in renewable electricity, (B) sensitivity of estimated syngas cost to electricity and CO_2_ prices, (C) comparison between coelectrolysis route and natural gas route to syngas in net CO_2_ emission. Note: R.E. (renewable electricity).

Technoeconomics is one of the decisive aspects in pushing research forward from the lab-scale to potential industrial applications, and the in-depth technoeconomics analysis in the initial development stage can help to find the bottlenecks from systematic perspectives. Figure 5A,B presents the technoeconomics of the modular coelectrolysis unit based on the above conceptual design for 1.0 kNm^3^/h syngas productivity. Some assumptions are described in the Supporting Information for micromonolithic cell operation. As shown in Figure 5A, the SOEC stack accounts for the highest share of the capital cost, approximately 73.18%, followed by the SOEC stack furnace (9.09%), and the complementary equipment cost accounts for only less than 20%. Therefore, reducing the cost of SOECs is critical for reducing capital costs; thus, highly efficient and cost-effective SOECs, such as micromonolithic cells, are extremely important.

For the operational cost, there is only renewable electricity and captured CO_2_ and water. The key assumption of the base case is to assume achievable 75 $ per metric ton in captured CO_2_ via large-scale disruptive DAC technology deployment in the future,^48^ 0.20 $/kWh in renewable electricity, and 0.50 $ per metric ton in water.^49^ It is obvious that electricity is the main cost source, accounting for 82.75%, and the CO_2_ cost is small at only 5.79%. Moving forward to seeing the cost breakdown of CAPEX and OPEX in Figure 5A, we can find that CPAEX (3.37%) is marginal compared to notable OPEX (96.63%). This key finding suggests that SOEC cost reduction or novel device design is not important, and the key driver for implementing sustainable syngas production in the current economy is the price of renewable electricity. However, the renewable electricity price is expected to decrease to $0.01/kWh, and the CO_2_ price is projected to decrease to less than $75 per metric ton in the near future or in RE-source places.^47,50^ The OPEX share will decline to 70%, and CAPEX will account for 30%. In this case, the SOEC design will no longer be marginal; rather, it will be the critical element for improving the technoeconomics of the syngas production process.

Based on the above analysis, it is important to understand the sensitivity of the syngas price to some key factors. Figure 5B shows the sensitivity of the predicted syngas price in the conceptual syngas production process. Obviously, the syngas price will decrease from approximately 1.0 $/Nm^3^_syngas_, which is still competitive with the biogas route (predicted 1.23–1.67 $/Nm^3^_syngas_ at 0.09 $/kWh electricity),^51^ to 0.10 $/Nm^3^_syngas_, which is closer to the current syngas market price of approximately 0.1 $/Nm^3^_syngas_ through fossil fuel routes.^52^

#### 3.2.3 Life Cycle Assessment

Life cycle assessment is a very important tool to provide quantitative information from the system level for identifying the key limiting points in achieving sustainability goals, and thus the strategies for further improvement can be formulated. Figure 5C presents the comparative carbon emissions in syngas production between the coelectrolysis route based on a micromonolithic SOEC and the conventional natural gas route via a gate-to-gate life cycle assessment approach. Obviously, the carbon footprint of syngas production is more sensitive to carbon emissions per unit of electricity than that of natural gas production. The natural gas route always has net positive carbon emissions and relatively stable carbon emissions over a large range of electricity emissions. However, the coelectrolysis route results in a wide range of net negative CO_2_ emissions. Considering the case of 100% renewable electricity (i.e., zero carbon emission) for the coelectrolysis route, it can produce a significant positive environmental impact, namely, −0.92 kgCO_2_/kg_syngas_. Even in the next decade, the electricity source will be a mix of fossil fuel and renewables, and the coelectrolysis unit will always have net negative carbon emissions when the electricity carbon emissions are less than 0.11 kgCO_2_/kWh; additionally, the coelectrolysis unit will continue to be competitive with the natural gas route in terms of environmental impact if the electricity carbon emissions are less than 0.3 kgCO_2_/kWh.

In summary, TEA and LCA prove the cost-effectiveness of micromonolithic coelectrolysis units and have a significant positive impact on sustainability. In the above analysis, there are no considerations of governmental incentives (e.g., carbon taxes or profit tax cuts) for negative carbon emission technologies. Therefore, the total cost has the potential to be further reduced, and the use of micromonolithic coelectrolysis units for syngas production will be more promising and competitive.

### 3.3 Section III. Comparison to Conventional Designs

Similar to the membrane design depicted in Figure S14, SOECs have almost the same design types, e.g., planar,^20^ spiral wound,^53^ tubular,^26^ and microtubular. Therefore, the key features, including the scaling up strategy and corresponding packing density, in membrane module design can be applied to SOEC stack design. Table S2 presents the typical features of the various membrane module designs. Obviously, the capacity of the microtubular design is several times greater than that of the planar and spiral wound designs and 1–2 orders of magnitude greater than that of the conventional tubular design, and the micromonolithic design further increases the volumetric capacity. This is mainly attributed to their characteristic dimensions (a diameter less than 1.0 mm for microtubules and several millimeters for others). Based on the current market for membranes, microtubular design has a relatively medium cost compared to other methods. Indeed, there are some less satisfactory points in microtubular design, typically in less-mature scale-up and low to medium mechanical strength compared to others. However, these aspects can be improved by adopting a micromonolithic design (which has better mechanical strength than a one-channel microtubular design) and well addressed by a suitable packing design, as discussed in Section I. Potentially, this design can be moved to a higher TRL and has fast deployment due to its superior technoeconomic-environmental performance. Additionally, due to its excellent performance, the potentially lowest land use can benefit the sustainable development goals of the United Nations and produce remarkable environmental benefits.

To more quantitatively compare different SOEC designs, we also conducted CAPEX estimation for conventional tubular and planar designs under the same standards. The key assumption, which is different from that of the micromonolithic design, is the current density. Herein, we assume −0.7 A/cm^2^ for conventional designs rather than −1.0 A/cm^2^ for micromonolithic design due to the clear consensus in electrochemical performance differences, as shown in Section I. Table S3 presents the summarized comparative data for various design types, and the detailed calculations can be found in the Supplementary Excel document. Obviously, to achieve the same syngas productivity, the micromonolithic design has the lowest stack cost, which is 23% lower than that of the planar design and approximately 89% lower than that of the tubular design. The cost of the conventional tubular design being almost 1 order of magnitude greater than that of the micromonolithic design is mainly attributed to the greater amount of raw materials required for SOEC cell manufacturing and the higher cost of stack furnaces due to the larger stack volume. It is not surprising to see that the planar design may present a lower unit cost because the planar design is more mature and the state-of-the-art microscale design is used for cost estimation.^54^ However, considering the optimization design from the discussion in the mechanical strength section, approximately 30% fewer materials for the optimal micromonolithic design can be saved. Thus, the optimal design for micromonolithic cells could achieve the lowest unit cost, which is ∼23% lower than that of advanced planar cells. Notably, from the perspective of environmental impacts, a less efficient SOEC design (namely, a lower current density) will undoubtedly cause more land use and more raw materials and, thus, cause higher carbon emissions throughout the whole life cycle. Finally, it is worth noting that the cost comparison does not provide precise values for each type of design since the material cost and other aspects will change over time or be affected by other undefined factors. However, the scale in terms of cost and particularly the ratio is reliable and will provide useful guidelines for selecting suitable technologies for specific application cases.

## 4 Conclusions

In summary, we conducted comprehensive studies on innovative micromonolithic SOEC cell design and sustainable electrification process design for the direct conversion of CO_2_ and H_2_O to valuable syngas. The micromonolithic cell design assisted by a controllable multistep manufacturing process enables an ultrahigh volumetric current density of −11.7 A/cm^3^ (4387 N m^3^_syngas_/h/m^3^) together with excellent long-term stability, which lays the foundation for a highly cost-effective, compact, modular electrolysis unit for the syngas process. Comparative analysis of various cell designs (planar, tubular, and micromonolithic) further confirmed the superior performance and emphasized that compared with conventional designs, micromonolithic designs can achieve up to 0.3–9 times lower costs and significant environmental benefits, including carbon emission and land use. TEA and LCA of the proposed conceptual process revealed that electricity is the dominant factor in the production of OPEX and SOEC cells in CAPEX, and thus, proper, flexible location selection (e.g., seaside) close to low-cost renewable electricity assisted by the merits of modular electrolysis units is the other key to cost-effective syngas production and process sustainability. Finally, this micromonolithic design can be straightforwardly extended to other electrochemical cells, and the conceptual syngas process at the seaside will enrich and inspire the discussion of a sustainable revolution in syngas production.

## Data Availability

All the data in this research are available upon reasonable request to the corresponding author.

## Supporting Information Available

Document S1: Supplementary Figures, Tables, and some details in TEA and LCA (PDF)
Document S2: Excel spreadsheets include original data and calculation for TEA (XLSX)
Document S3: Excel spreadsheets include original data and calculation for LCA (XLSX)
