# Supplementary material for: Micromonolithic Electrochemical Cells for Sustainable Syngas Production from H2O and CO2
Source: ACS Sustain Chem Eng. 2025 May 5;13(19):7005–16. doi: 10.1021/acssuschemeng.4c10889 (PMC12093367; doi:10.1021/acssuschemeng.4c10889)
Supplement: Supplementary file 1 — sc4c10889_si_001.pdf [file sc4c10889_si_001.pdf]

Electronic Supplementary Information

Micromonolithic Electrochemical Cells for Sustainable Syngas Production from H_2O and CO_2

Peng Yan[1], Tao Li[2], Kang Li[1]*

[1] Barrer Centre, Department of Chemical Engineering, Imperial College London, SW7 2AZ, London, United Kingdom

[2] MOE Key Laboratory of Energy Thermal Conversion & Control, School of Energy and Environment, Southeast University, Nanjing 210096, China

* Corresponding author: kang.li@imperial.ac.uk (Kang Li)

ORCID: https://orcid.org/0000-0002-8096-8622 (Peng Yan)

Number of pages: 29
Number of figures: 14
Number of schemes: 2
Number of tables: 7

Table of Contents

1. Section S1: experimental methods S3
2. Section S2. Supporting data for R&D section in the manuscript S7
3. Section S3. Section S3. TEA method details S24

Section S1. Experimental methods

Materials: Powders of inorganic materials, including 8 mol% Yttria-stabilized zirconia (YSZ8), nickel oxide (NiO), and lanthanum strontium manganite ($(La_{0.8}Sr_{0.2})_{0.95}MnO_{3-\delta}$), were purchased from FuelCell Materials (USA). Polymethyl methacrylate (PMMA) (Acrypet™, Mitsubishi Chem. Co., JP), Arlacel P135 (Uniqema), N-methyl-2-pyrrolidone (NMP, GPR Rectapur, VWR), and ethanol (HPLC, VWR) were used as the binder polymer, dispersant polymer, solvent and solvent, respectively.

SOEC cell fabrication: Full SOECs were fabricated by a combined controllable phase inversion and sintering method. **(1) YSZ8-NiO electrode:** The 6-channel YSZ8-NiO electrode precursor was fabricated by a phase inversion-assisted extrusion process, similar to multichannel hollow fiber spinning, as described previously[1,2]. The spinneret has 6 orifices (I.D.=0.8 mm, O.D.=1.1 mm) arranged symmetrically inside a circular space with the I.D. 4.0 mm, their central distance is around 1.3 mm to ensure the 0.2 mm gap between two adjacent orifices, and the needle center to the center of spinneret is around 1.3 mm. Briefly, NiO, YSZ8, NMP, dispersant and PMMA were mixed, ball-milled, and roll-milled to obtain a stable suspension with a homogeneous dispersion of NiO and YSZ8 particles. The suspension was degassed for 3-6 hours in a vacuum system to eliminate all the air bubbles in the suspension. The resulting suspension was transferred to a 200 ml stainless-steel syringe for spinning with a custom-designed 6-bore tube-in-orifice spinneret. A mixture of ethanol (30 wt%) and DI water (70 wt%) was used as the internal bore liquid, and tap water was used as the coagulation bath during the spinning process. The as-obtained 6-channel YSZ8-NiO electrode precursor was straightened, dried and presintered for 3 hours at 1150 °C. **(2) YSZ8 electrolyte coating:** YSZ8 solution in ethanol was dip-coated to the above YSZ8-NiO electrode at a 2 mm/s withdrawal speed. The YSZ8|YSZ8-NiO precursor was sintered for 6 hours at 1400 °C to obtain a dense and robust electrolyte. **(3) For the oxygen electrode coating,** LSM and YSZ8 at 50 wt%, 50 wt%, or pure LSM powder were dispersed in ethylene glycol by 2 days of ball milling to obtain homogeneous inks. The LSM-YSZ8 and LSM inks were brush-painted onto YSZ8|YSZ8-NiO in sequence, followed by sintering for 2 hours at 1100 °C. Finally, full SOECs LSM/LSM-YSZ8|YSZ8|YSZ8-NiO were prepared.

Table S1. Suspension composition and fabrication conditions of the YSZ8-NiO electrode

Suspension composition	Ceramics (wt%)	63.0 (NiO: YSZ=3:2)	
	NMP (wt%)	28.0	
	PMMA (wt%)	8.4	
	Dispersant (wt%)	0.6	
		Normal Electrodes (Configuration 1)	Thinner Electrodes (Configuration 2)
Fabrication conditions	Extrusion rate (ml/min)	9	9
	Bore liquid rate (ml/min)	13	16
	Air gap (mm)	5	5

Characterization: (1) Geometrical morphology: The morphology of the micromonolithic SOECs was characterized using a scanning electron microscope (SEM, Zeiss Sigma300). **(2) Gas-tightness:** The gas-tightness of the YSZ8 electrolyte coating was tested using an N_2 permeation method as described in our previous study [3], where a digital pressure gauge was adopted to monitor the pressure of the gas permeation setup instead, and a sintering condition with a fully dense YSZ8 electrolyte was obtained based on a gas-tightness test (a negligible drop in pressure during the 23-hour test period). The only difference is in the calculated membrane area, which is calculated by the outer diameter for multichannel samples in this paper rather than by the outer and inner diameters for single-channel samples. The gas permeance is calculated based on the cylinder pressure change with time as shown below:

$$\mathrm{P}_{per} = \frac{V}{R \cdot T \cdot A_m \cdot t} \ln\left(\frac{P_0 - P_a}{P_t - P_a}\right)$$

where P_{per} is the permeance of the test membrane (mol/m^2/Pa/s); V is the volume of the test cylinder (m^3); R is the gas constant (8.314 J/mol/K); and T is the measured temperature (K). P_0 and P_t are the initial and final measured pressures in the test cylinder (Pa), respectively; P_a is the atmospheric pressure (Pa). A_m (m^2) is the membrane area, where $A_m = \pi \cdot D \cdot L$. D is the outer diameter of the cell (m). L is the effective length of the cell (m), and t is the time for measurement (s). **(3) Mechanical strength:** The mechanical strength of the micromonolithic SOECs was measured using a tensile tester (EZ 50) with a load of 100 N via a three-point bending method. The samples were placed on two lower sample holders with a span distance of 3.0 cm, and 3 samples were tested to determine the average mechanical strength. The bending strength (δ_F) was calculated based on the following equation:

$$\delta_F = 8FLD_o/(D_o^4 - D_i^4)$$

where F is the measured force when the micromonolithic samples are broken, N; L, D_o, and D_i represent the characteristic length (3.0 cm), outer diameter and estimated inner diameter (rounded from 6 channels to 1 channel based on the cross-sectional area), respectively.

(4) Porosity measurement: The as-prepared cell after H_2 reduction is characterized by mercury intrusion porosimetry method (Autopore IV 9500, Micromeritics) under the pressure from 0.10 to 60000 psia. The typical sample amount is around 0.5g and it is worth mentioning that the cell sample was broken, and the central part of the cell is removed because the central part is not that relevant to the electrolysis and the most important part is the peripheral part of the cell.

Electrochemical measurements: Three aspects are presented to fully describe the electrochemical measurements of the micromonolithic SOECs. **(1) Assemble the full SOEC cell:** A silver wire 0.25 mm in diameter (AG5485, Advent Research Materials, UK) was coiled onto the LSM/LSM-YSZ8 electrode and YSZ8-NiO electrode, and the contact between the electrode and the silver wire was enhanced by silver paste (RS Company, UK). Subsequently, the cell was fixed into two gas-tight alumina tubes and sealed with ceramic adhesive (Ceramabond 552-VFG, Aremco, USA). The cell was then assembled into a quartz tube with custom-made Swagelok fittings, where the silver wires were removed for further connection to an electrochemical workstation. **(2) Electrochemical evaluation system:** The fully assembled cell was connected to an electrochemical workstation (Potentiostat/Galvanostat, Iviumstat, NL) with a 4-electrode method, and the gas effluent from the SOEC cell was evaluated by GC with a TCD detector (Varian GC-3900). The experimental system for evaluating the SOEC is illustrated in ***Figure S1***, where the furnace for hosting the SOEC cells is ignored for visual display. The heating zone of the furnace was 30 cm in length, and it was calibrated by a 1.0 mm K-type thermocouple between 600 and 900 °C. The isothermal zone is approximately 4.0 cm long, which is long enough for the electrolysis cell test. **(3) Cell running details:** The measurements were conducted at 700, 750, and 800 °C, with a 160 ml/min air flow rate on the shell side and a 100 ml/min gas mixture (CO_2, H_2O) on the lumen side for the electrolysis mode and 60 ml/min H_2 for the fuel cell mode. Before the measurement, the cell was heated to 600 °C with a 2 °C/min ramping rate under Ar gas at the lumen side and then switched to 10% H_2

in an Ar atmosphere overnight reduction at 600 °C. After that, the cell was further heated to the target test temperature with a 2 °C/min ramping rate for a series of current-voltage (IV) measurements, electrochemical impedance spectroscopy (EIS), long-term stability measurements and gas product analysis. EIS was conducted in the frequency range of 10^5~0.01 Hz with a signal amplitude of 10 mV under open-circuit conditions (OCV). IV curves were measured at the relevant voltage range starting from the OCV with a step size of 5 or 10 mV and a scan rate of 5 mV. **(4) Cell test for configurations #1, #2, and #3:** Typically, three samples are evaluated for their electrochemical performance, and the deviation is generally within ±10%, which proves excellent cell reproducibility. The temperature was monitored by a 1.0 mm K-type thermocouple attached to the outer wall of the quartz tube.

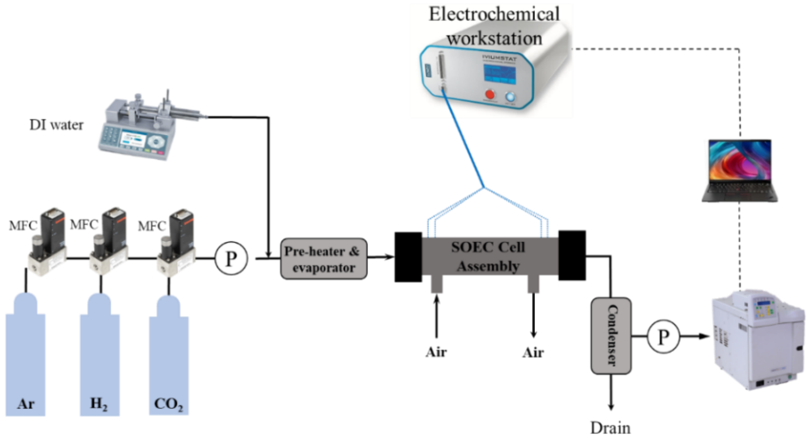

Figure S1. Schematic diagram of the experimental setup for evaluating micromonolithic SOECs.

Techno-economics (TEA): Techno-economics was conducted with classical chemical engineering methodology; here, the factorial methodology, specifically the hand method, was adopted [4,5]. A detailed description of the TEA methodology, data, and assumptions is provided in **Section S3** of this supplementary document. The data and calculation processes, including the comparison of data among micromonolithic, conventionally tubular, and planar cell types, are described as well.

Life cycle assessment (LCA): Environmental impact, in terms of CO_2 emission, was assessed for the electrolysis subsystem with a predefined system boundary, as shown in **Scheme 2,** by focusing on the gate-to-gate scope (only focusing on the manufacturing processes and syngas production process in this case) [6-8], and the reference data for the natural gas route to syngas were extracted from the literature [7].

Section S2. Supporting data for R&D section in the manuscript.

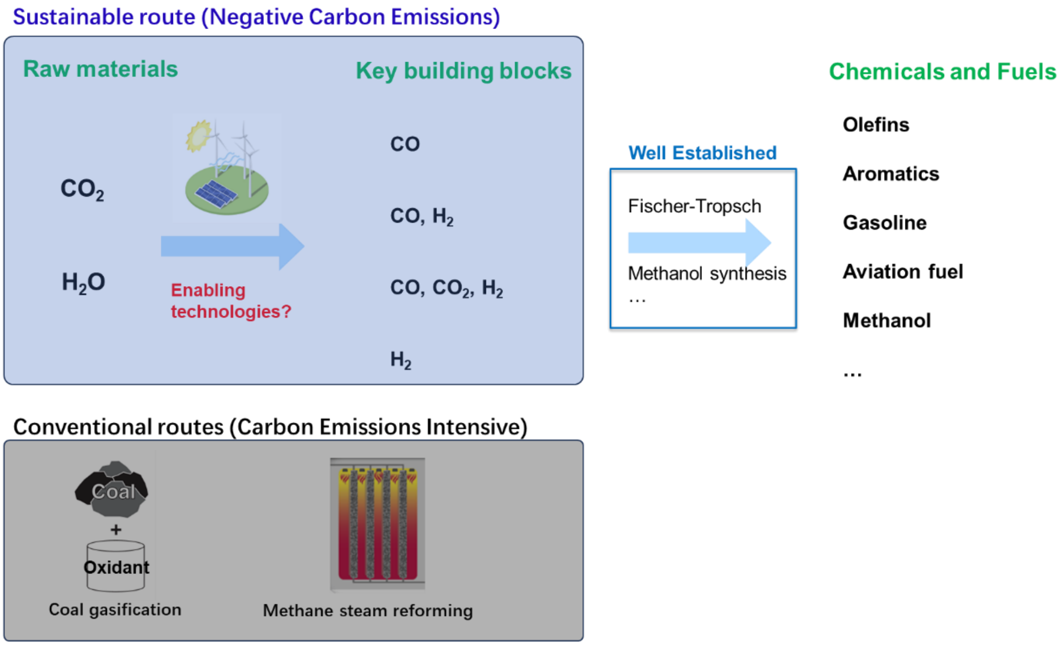

Scheme S1. The broad view of the chemical industry involving syngas production and conversion.

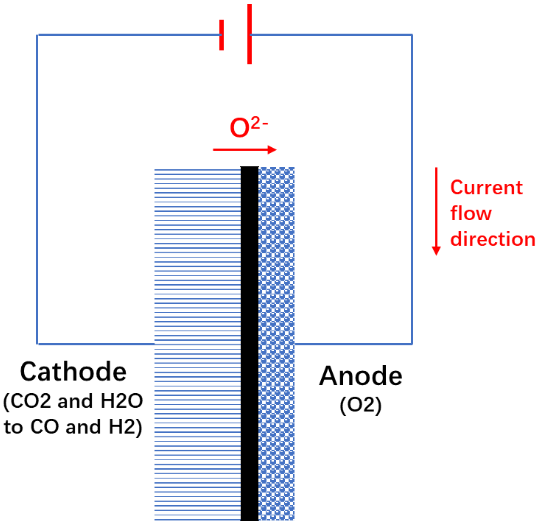

$$2CO_2 \rightarrow 2CO + O_2$$

$$2H_2O \rightarrow 2H_2 + O_2$$

(A)

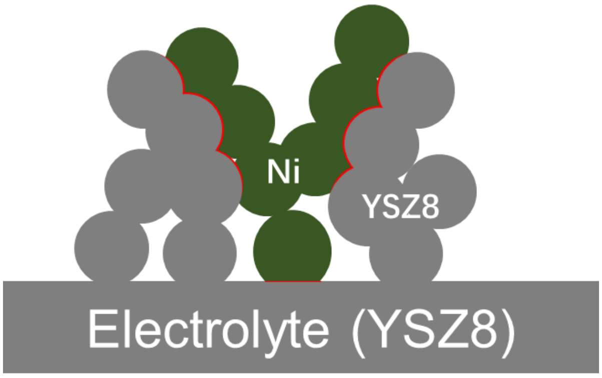

(B)

Scheme S2. Illustration of the SOEC process: (A) involved reactions for syngas production in SOEC, (B) the active sites place (i.e. triple-phase boundaries) in Ni-YSZ8 electrode, shown in red.

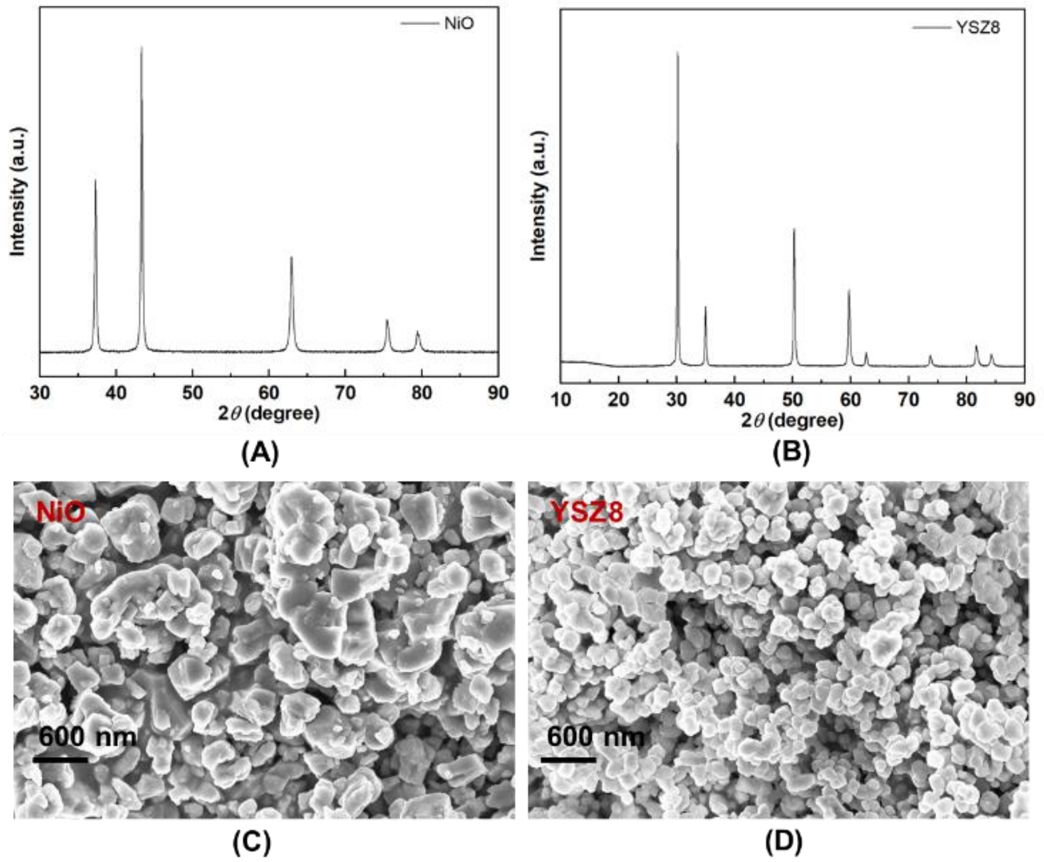

Figure S2. Characterization of electrode materials (NiO, YSZ8)

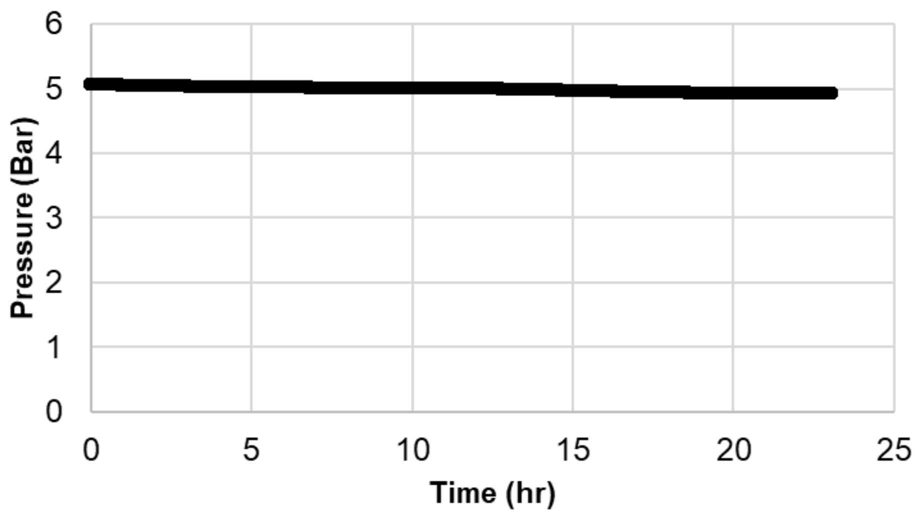

(A)

V	0.0003	m^3	L	4	cm
R	8.314	J/mol/K	D	2.6	mm
T	293.15	K	P_0	5.06	bar
A_m	0.0003266	m^2	Pt	4.91	bar
t	83126	s	Pa	1.01	bar
$\ln(P_0\text{-Pa})/(\text{Pt-Pa})$	0.03774				
$\mathbf{P_{per}}$	**1.7113E-10**	**mol/m²/Pa/s**			

(B)

Figure S3. Gas-tightness experimental test. (A) Cylinder pressure trend with time. (B) Permeance of the electrolyte-coated half-cell YSZ8|YSZ8-NiO (the permeance is $1.71 \cdot 10^{-10}$ mol/m^2/Pa/s, which is negligible).

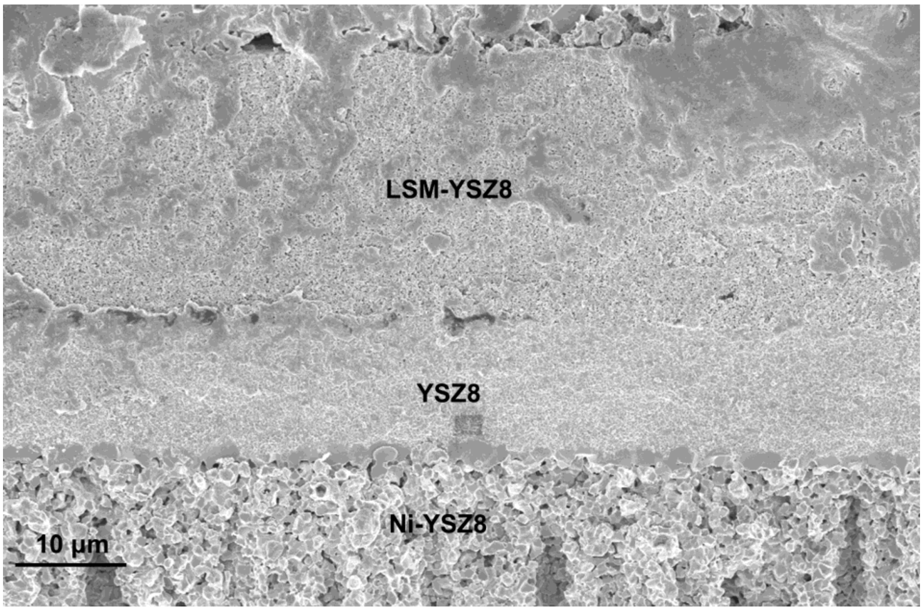

Figure S4. SEM shows the interfaces status of the as-prepared cell before durability test

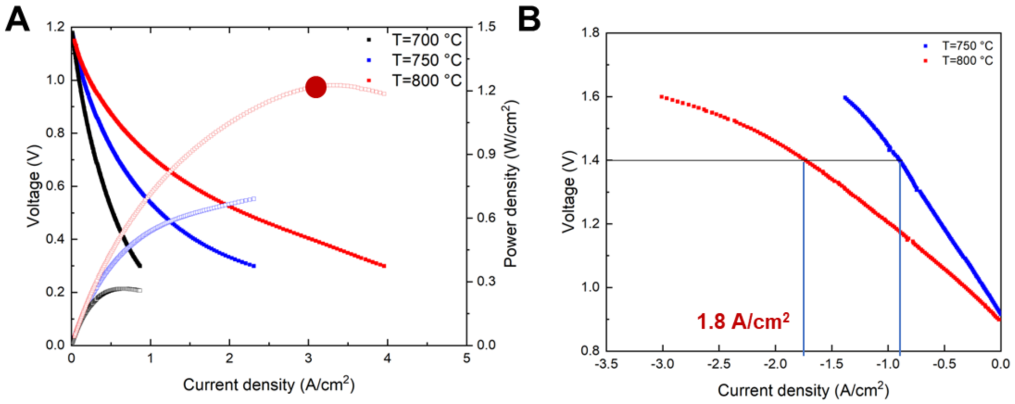

C

T/℃	700	750	800
Mass transfer resistance (Ω·cm^2)	1.62	1.25	1.23

Feed: 45 sccm H_2O, 27.5 sccm CO_2, 27.5 sccm H_2

Figure S5. Performance of basic hollow fiber electrode design (configuration #1) under fuel cell mode and electrolysis mode, including the analysis of mass transfer resistance.

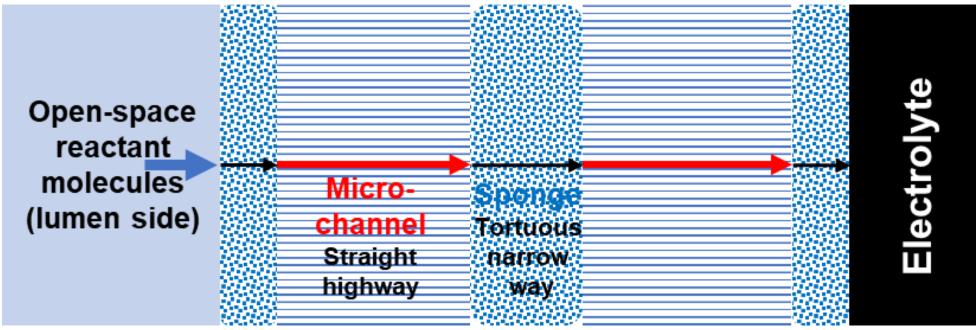

(A)

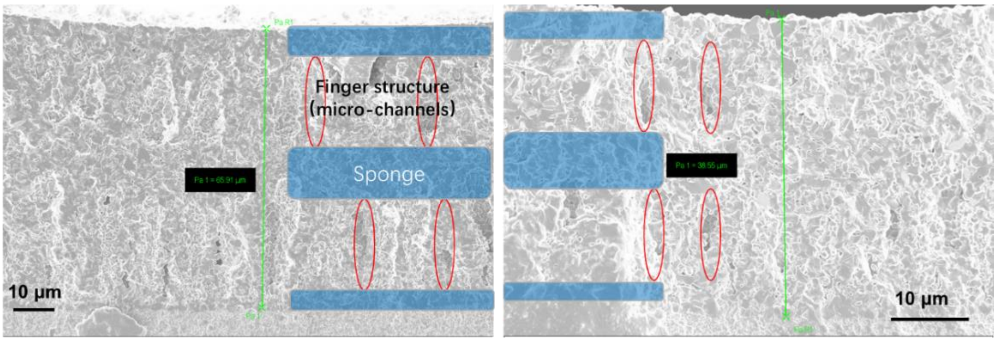

(B)

Figure S6. (A) Schematic illustration of the mass transfer process of gas going through the wall of a hollow fiber electrode. (B) The cross-sectional geometries of micromonolithic walls for Configuration #1 (left) with a thickness of 65 μm and Configuration #2 (right) with a thickness of 38 μm. Configuration #2 has a shorter finger structure and contains fewer sponges than does Configuration #1.

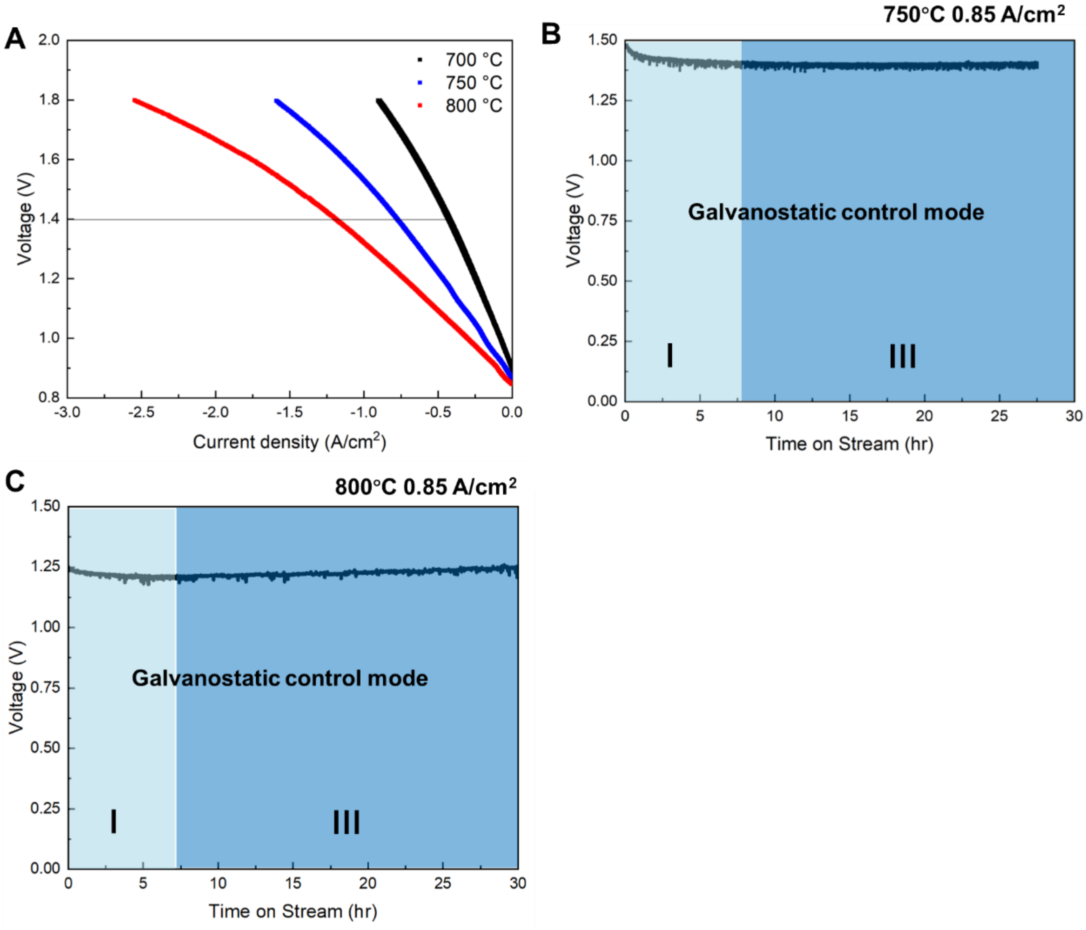

Figure S7. Cell performance and short-term stability under the galvanostatic control mode with a higher CO_2 concentration feed at (B) 750 °C and (C) 800°C (feed: 10 sccm H_2, 45 sccm CO_2, 45 sccm H_2O).

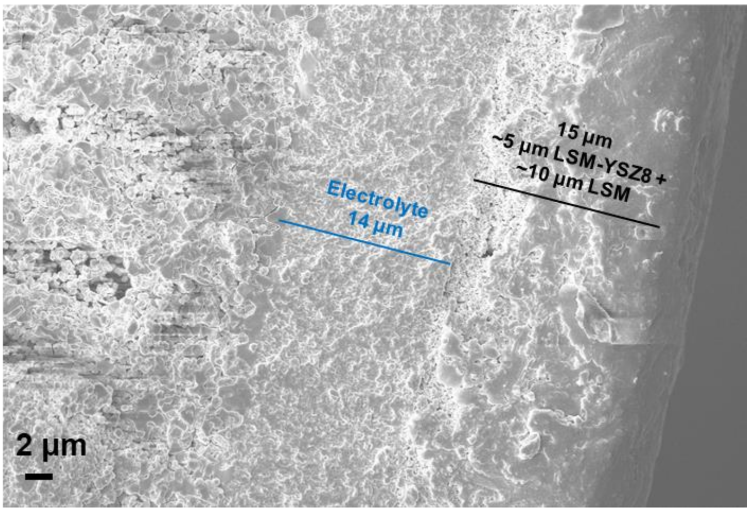

Figure S8. An improved design (configuration #3 in Figure 3) for better long-term stability was achieved by deliberately increasing the overall polarization resistance and decreasing the number of active sites available for the catalytic reaction.

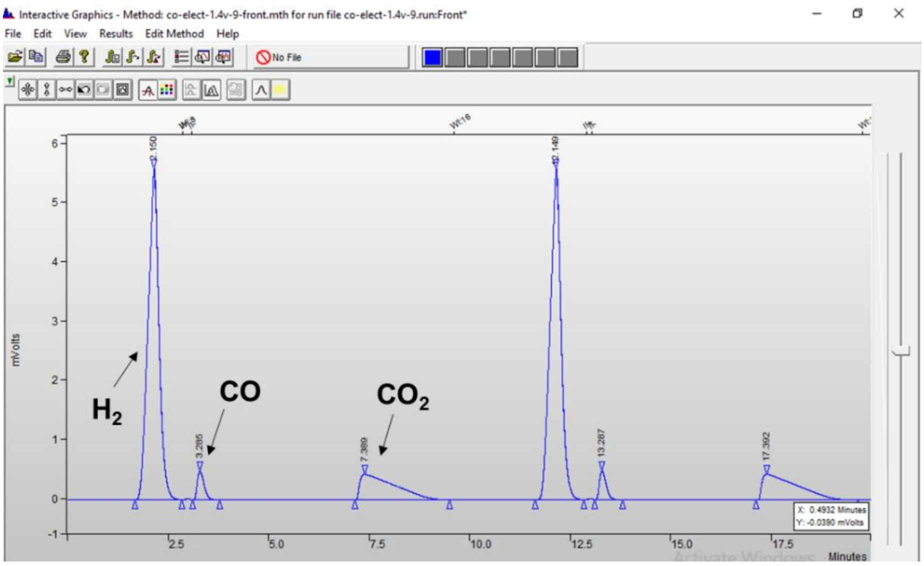

Figure S9. The typical GC sampling data for the effluent analysis from the SOEC during the long-term stability test (two sample data in one document).

Faraday Efficiency:

$$\mathrm{FE} = \frac{\mathrm{V}_{prod} \cdot P \cdot N_A \cdot 2}{R \cdot T \cdot I \cdot A \cdot t \cdot Col}$$

Where, V_{prod}: generated H_2 and CO by co-electrolysis process, ml/min; P: gas pressure, Pa; N_A: $6.02*10^{23}$ mol^{-1}; R: gas constant, 8.314 J/mol/K; T: temperature, K; I: current density, A/cm^2; A: active area, cm^2, t: time, second; Col: $6.25*10^{18}$, electron per coulomb.

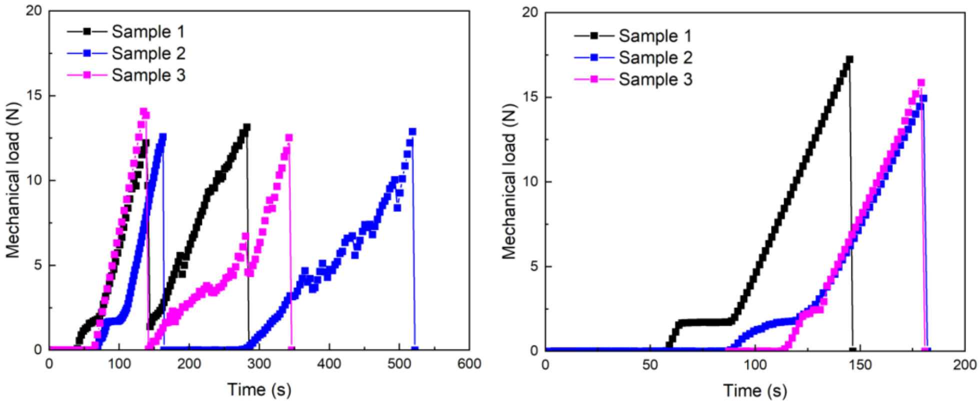

(A) (B)

Figure S10. Raw data to present the mechanical behavior of micromonolithic SOECs. (A) Two-stage breaking behavior of cells (YSZ8-NiO) without an electrolyte layer. (B) One-stage breaking behavior of cells (YSZ8|YSZ8-NiO) with an electrolyte layer.

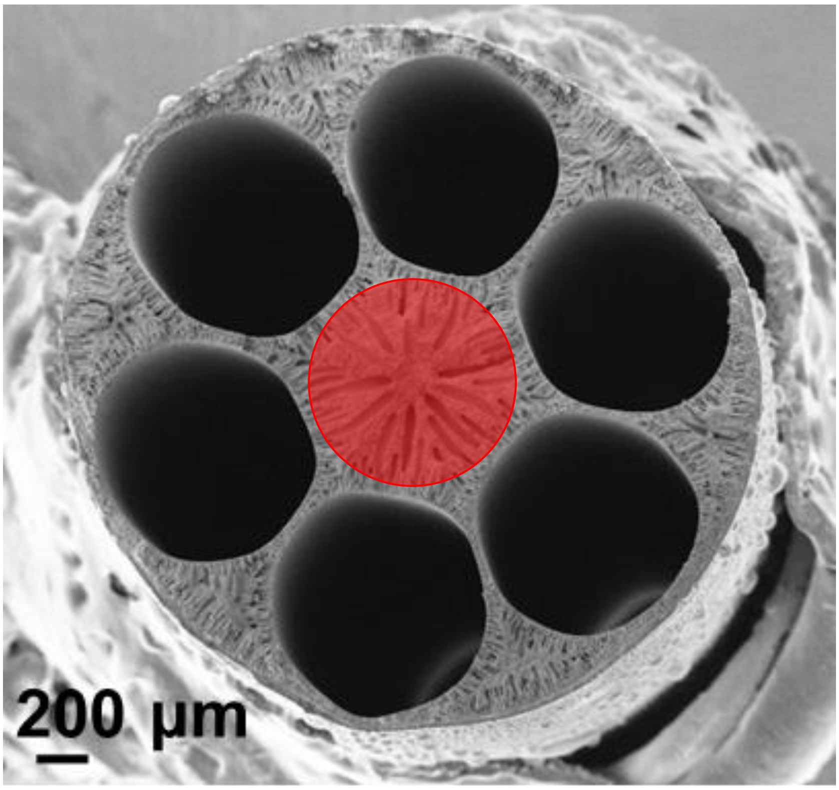

Figure S11. Red-zone percentage (approximately 30%) in the solid-phase cross section

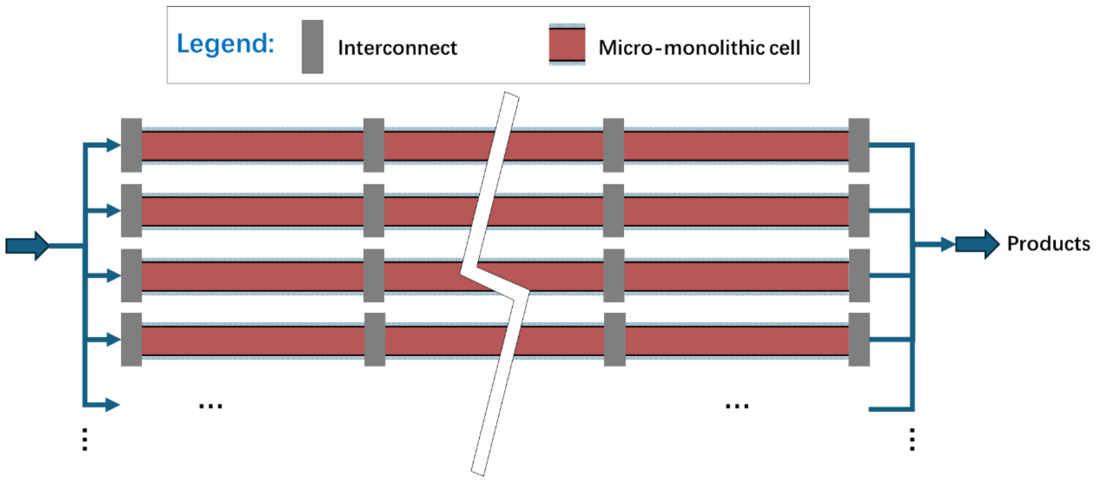

Figure S12. The scaling-up concept of parallel-series configuration.

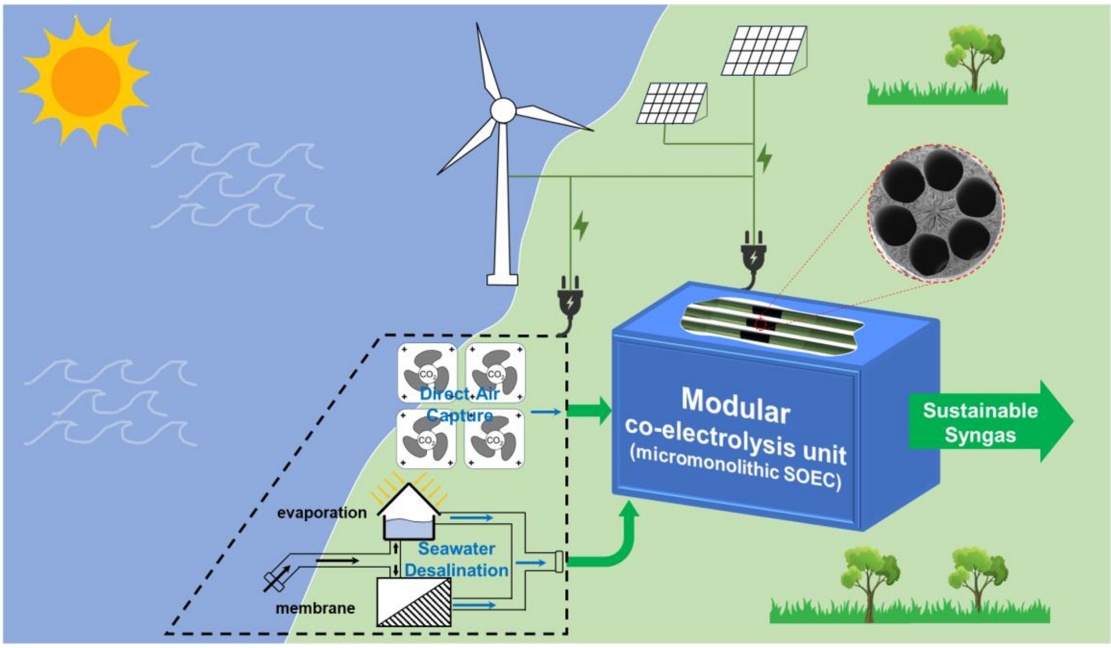

Figure S13. Enlarged picture of modular syngas production unit shown in Scheme 2B.

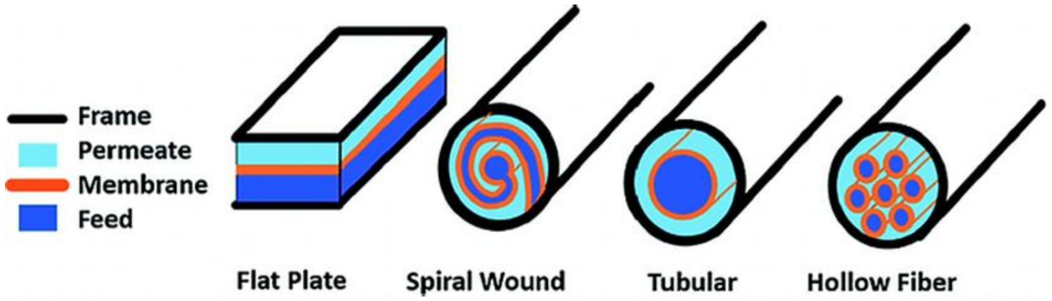

Figure S14. Typical membrane module types. The hollow fiber type is also called the microtubular type. A micromontolithic (microtubular monolithic) design is an advanced type of microtubular design since it provides a much greater volumetric surface area and better mechanical strength. Taken from an online source (https://www.molecularforcesllc.com/post/an-introduction-to-membrane-modules).

Table S2. Typical membrane module types and their key features

Type	Characteristic dimension	Capacity (m^2/m^3)	Cost level	Mechanical strength	Scale-up	TRL	Land use
**Planar or Spiral wound**	1-2 mm thickness	200-800	Low	Medium	Easy	9	Medium
**Tubular**	5-15 mm	30-200	High	High	Medium	9	High
**Hollow Fiber (microtubular)**	<1.0 mm	500-9000	Low /Medium	Medium /Low	Difficult	Polymeric (9) Ceramic (6-7)	Low

Note: Micromonolithic (microtubular monolithic) design is an advanced form of microtubular design since it provides a much greater volumetric surface area and better mechanical strength. Therefore, the innovative micromontolithic design has better features than the hollow fibers (microtubular) presented in the table.

The detailed calculation and comparison among these three different cell designs for SOECs are presented in supplementary Excel document.

Table S3. Comparison of the stack and unit costs for micromonolithic (abbreviated as HF), planar and tubular designs for defined syngas productivity.

**Stack type**	**HF[base]**	**HF[opti]**	**Planar[a]**	**Tubular[design 1]**	**Tubular[design 2]**
Stack cost (million $)	0.41	0.29	0.53	3.84	3.27
Unit cost ($\$/m^2$)	1541	1078	1401[b]	10116	8613

[a] The cathode is made of a NiO-YSZ8 functional layer (10 μm) and a NiO-YSZ3 supporting layer (300 μm).

[b] The estimated unit cost is in line with the cost estimation in the literature [9].

[Design 1]: The cathode is made of NiO-YSZ8 (1.50 mm in thickness)

[Design 2]: The cathode is made of a NiO-YSZ8 functional layer (10 μm) and a NiO-YSZ3 supporting layer (1.49 mm in thickness).

[base]: the basic design (cross-section with large central but useless part), as shown in **Figure 1** in the main text

[opti]: the optimal design (eliminating the central part as discussed in the mechanical strength analysis): (100-30)% = 70% base HF case cost as estimated material savings in Figure S8.

The detailed calculation and comparison among these three different cell designs for SOECs are presented in supplementary Excel document.

Section S3. TEA method details

Table S4. Basic geometrical and operation parameters of the micromonolithic cell stack

Cell O.D. (mm)	Operation performance (A/cm^2)	Cell stack configuration	Center distance (mm)	Operation Temperature (°C)	Operation pressure (atm)	Lifetime (hrs)
2.60	1.0	Square	5.20	750~800	1	43000

Note: Herein, we use the smallest packing density for the calculation.

Basic assumptions:

1. The lifetime is 5 years with 8600 hrs of operation per year for continuous centralized applications, while the lifetime is 10 years with 4300 hrs of operation per year for intermittent decentralized applications considering the intermittency of renewable electricity. The total lifetime for each case is 43000 hrs.
2. The length of a single SOEC cell is 12 cm (10 cm active domain, 2.0 cm end connection domain).
3. The silver wire length is 8.0 cm/cm for the active domain of the SOEC cell
4. The silver paste volume was 2.0 cm for each SOEC.

Modular design:

The required SOEC area or volume was calculated based on the 1.0 Nm^3/hr production capability of a single module. For the below capital cost and the operational cost, a 1000 Nm^3/hr production capability, which is more realistic in practice, is used.

1. V_{gas}=1.0 Nm^3/hr production capability for a single module, and the needed SOEC cell surface area is:

$$Q_{eff} = A \cdot I \cdot t \cdot FE$$

$$Q_{eff} = {V_{gas}}/{a} \cdot n \cdot F$$

$$A = V_{gas}/a \cdot n \cdot F/(I \cdot t \cdot FE) = \mathbf{2659\ cm^2}$$

where Q_{eff} is the effective charge transferred to the syngas molecule, I is the current density, t is time, FE is the Faraday efficiency, V_{gas} is the produced gas volume in the STP, a is the gas constant (22.4 L/mol), n is the number of transferred electrons per syngas molecule, and F is the Faraday

constant, 96485 C/mol. Here, FE=90% is assumed to include the consideration of any possible system leakage, although coelectrolysis is deemed to have a 100% Faraday efficiency.

2. The required volume per cm^2 of active domain:

$$V_{cell}=5.20/10*5.20/10*1/0.82\ cm^3=0.33\ cm^3$$

3. Module volume for 1.0 Nm^3/hr production capability

$$V_m=2659*0.33*(12/10)=\mathbf{1053\ cm^3}$$

In summary, the modular design rule is approximately **950 $Nm^3/hr/m^3_{cell\_module}$, or 42.4 $kmol/hr/m^3$** (If the SOECs are in closer packing, namely, the cells contact each other, the volumetric productivity of the modular unit is 3800 $Nm^3/hr/m^3$, or 169.6 $kmol/hr/m^3$ for the square-packed stack, **4387 $Nm^3/hr/m^3$**, or 195.8 $kmol/hr/m^3$ for the triangular-paced stack (corresponding to 11.66 A/cm^3).

Further assumptions in TEA of the modular coelectrolysis unit:

1. Energy, including power and heat, which is fully based on renewable electricity, is derived from renewable wind and solar power.
2. The feedstocks are 50 mol% CO_2 + 50 mol% H_2O, which are fully from renewable and recyclable sources, e.g., CO_2 from CO_2 capture in power plants or direct air capture (DAC) and water from seawater desalination.
3. The equipment is 100% electricity powered, and the energy efficiency of effective heat and electrons is 90%, with 10% of the energy wasted in the environment due to imperfect heat insulation.
4. For the SOEC cell, the Faraday efficiency is 90%, including any possible system leakage, although coelectrolysis is deemed to have a 100% Faraday efficiency.
5. The theoretical energy requirement of coelectrolysis is simply estimated from steam electrolysis (249 kJ/mol) and CO_2 electrolysis (283 kJ/mol) thermodynamics, namely, 266 kJ/mol[10].
6. The coelectrolysis process results in 90% conversion and does not include separation cost.
7. Syngas is sent directly downstream without storage tanks in the coelectrolysis unit.
8. The productivity of the modular coelectrolysis unit is 1000 Nm^3/hr.
9. Electricity price: currently use 20 cents/kWh, while the future goal of renewable electricity is 1 cent/kWh

10. The airflow rate in the anode is 1.5 times the feedstock (CO_2 and steam) flow rate in the lumen side.
11. The energy for heating the air is 60% recovered.

OPEX (operating expenditure):

1. Energy cost:
   - ✧ CO_2 from R.T. to 800 °C
   - ✧ Water from R.T. to 800 °C
   - ✧ Air from R.T. to 800 °C
   - ✧ Electrolysis energy
2. Feedstock cost
   - ✧ CO_2 cost
   - ✧ Water cost
   - ✧ Compressed air
3. Maintenance cost

   It includes labor costs and system maintenance. The cost is estimated to be 10% of the energy and feedstock costs.

CAPEX (capital expenditure):

1. Overall method of estimating equipment cost

$$C_e = a + bS^n$$

where Ce is the cost of the specific equipment, a, b, and n are constants for the specific equipment, and S is the specific equipment size.

Table S5. Constant parameters of cost estimation for various equipment

Equipment	Units of Size, S	a	b	n
**Pump**	L/s	3300	48	1.2
**Heat Exchanger**	area, m^2	10000	88	1.0
**Boiler**	kg/h steam	4600	62	0.8
**Furnace**	duty, MW	7000	71000	0.8

2. Basics for estimating SOEC furnaces

The furnace is sized based on its power requirements to complete the heating of the SOEC stack, and the specific heat capacity of the cell materials is used for the estimation.

$$Q_{\text{Furnace}} = \sum_i C_{p,i} \cdot (T_{op} - T_{R.T.}) * m_i$$

where $C_{p,i}$ denotes the specific heat capacity of the raw materials, m_i denotes the mass of the raw materials, and T_{op} and $T_{R.T.}$ denotes the operation temperature and room temperature, respectively. The specific heat capacities of the various materials are listed below.

Table S6. The specific heat capacity of various materials

**Materials**	YSZ	NiO	LSM	Silver
**$C_{p,i}$, kJ/kg/K**	0.60	0.68	0.30	0.24

Note: 2 °C/min ramp rate

$$Q_{H.T.} = \sum_j C_{p,j} \cdot (T_{op} - T_{R.T.}) * m_j$$

where $C_{p,j}$ denotes the specific heat capacity of the raw materials, mj denotes the mass of the raw materials, and T_{op} and $T_{R.T.}$ denotes the operation temperature and room temperature, respectively.

$$LMTD = \frac{(T_{hot,in} - T_{cold,out}) - (T_{hot,out} - T_{cold,in})}{\ln((T_{hot,in} - T_{cold,out}) / (T_{hot,out} - T_{cold,in}))}$$

A temperature difference between the hot and cold streams of 100 and 50 °C was set for the countercurrent heat exchanger. Thus, the inlet and outlet temperatures of the hot stream are 900 °C and 75 °C, respectively.

The overall heat transfer coefficient (U) is set at 30 $W/K/m^2$ for gas–gas transfer.

$$A_{H.T.} = \frac{Q_{H.T.}}{U \cdot LMTD}$$

3. Auxiliary

It includes an operation control system, insulation, tubing, etc., and accounts for 10% of the capital cost of key equipment.

4. Hand factor

In the hand factor method, a specific factor is applied to the above-estimated equipment cost, and the final value is regarded as the total cost of the equipment, including shipping, on-site installation,

labor, indirect module expense, etc. [4]. The specific factors for each type of relevant equipment are listed below.

Table S7. Hand factors for various equipment

Equipment	Pump	Heat exchanger	Boiler	Furnace	SOEC stack
Hand factor	4	3.5	2	2.0	2.5

5. Total cost

$$C = F\sum Ce$$

where C denotes the total capital cost of equipment, C_e denotes the item cost of each piece of equipment, and F denotes the hand factor.

References:

1. Li T, Rabuni MF, Kleiminger L, Wang B, Kelsall GH, Hartley UW, Li K. A highly-robust solid oxide fuel cell (SOFC): simultaneous greenhouse gas treatment and clean energy generation. *Energy & Environmental Science.* 2016;9(12):3682-3686.

2. Li T, Lu X, Rabuni MF, Wang B, Farandos NM, Kelsall GH, Brett DJL, Shearing PR, Ouyang M, Brandon NP, Li K. High-performance fuel cell designed for coking-resistance and efficient conversion of waste methane to electrical energy. *Energy & Environmental Science.* 2020;13(6):1879-1887.

3. Tan X, Liu Y, Li K. Mixed conducting ceramic hollow-fiber membranes for air separation. *AIChE J.* 2005;51(7):1991-2000.

4. Towler G, Sinnott R. Chemical Engineering Design: Principles, Practice and Economics of Plant and Process Design. 5th ed. Oxford, UK2009.

5. van Amsterdam MF. *Factorial techniques applied in chemical plant cost estimation: a comparative study based on literature and cases*. Delft, The Netherlands: Department of Chemical Engineering, Delft University of Technology; 2018.

6. Tackett BM, Gomez E, Chen JG. Net reduction of CO2 via its thermocatalytic and electrocatalytic transformation reactions in standard and hybrid processes. *Nature Catalysis.* 2019;2(5):381-386.

7. Schreiber A, Peschel A, Hentschel B, Zapp P. Life Cycle Assessment of Power-to-Syngas: Comparing High Temperature Co-Electrolysis and Steam Methane Reforming. *Frontiers in Energy Research.* 2020;8.

8. Kleinekorte J, Fleitmann L, Bachmann M, Kätelhön A, Barbosa-Póvoa A, Assen Nvd, Bardow A. Life Cycle Assessment for the Design of Chemical Processes, Products, and Supply Chains. *Annual Review of Chemical and Biomolecular Engineering.* 2020;11(1):203-233.

9. Anghilante R, Colomar D, Brisse A, Marrony M. Bottom-up cost evaluation of SOEC systems in the range of 10–100 MW. *Int J Hydrogen Energ.* 2018;43(45):20309-20322.

10. Harrison K, Remick R, Hoskin A, Martin G. Hydrogen Production: Fundamentals and Case Study Summaries: Preprint2010.
